# Patterns of brain function associated with cannabis cue-reactivity in regular cannabis users: a systematic review of fMRI studies

**DOI:** 10.1007/s00213-021-05973-x

**Published:** 2021-09-10

**Authors:** Hannah Sehl, Gill Terrett, Lisa-Marie Greenwood, Magdalena Kowalczyk, Hannah Thomson, Govinda Poudel, Victoria Manning, Valentina Lorenzetti

**Affiliations:** 1 Neuroscience of Addiction and Mental Health Program, Healthy Brain and Mind Research Centre, School of Behavioural and Health Sciences, Faculty of Health Sciences, Australian Catholic University, Melbourne, Daniel Mannix building, 17 Young Street, Fitzroy, VIC 3065 Australia; 2 Research School of Psychology, Australian National University, Canberra, Australia; 3 The Australian Centre for Cannabinoid Clinical and Research Excellence (ACRE), New Lambton Heights, New South Wales Australia; 4 Mary Mackillop Institute for Health Research, Australian Catholic University, Melbourne, Australia; 5 Turning Point, Eastern Health, Monash University, Melbourne, Australia

**Keywords:** Cannabis, Craving, Functional magnetic resonance imaging, fMRI, Cue-reactivity, Neuroimaging, Brain

## Abstract

**Rationale:**

Regular cannabis use (i.e. ≥ monthly) is highly prevalent, with past year use being reported by ~ 200 million people globally.High reactivity to cannabis cues is a key feature of regular cannabis use and has been ascribed to greater cannabis exposure and craving, but the underlying neurobiology is yet to be systematically integrated.

**Objectives:**

We aim to systematically summarise the findings from fMRI studies which examined brain function in cannabis users while exposed to cannabis vs neutral stimuli during a cue-reactivity fMRI task.

**Methods:**

A systematic search of PsycINFO, PubMed and Scopus databases was pre-registered in PROSPERO (CRD42020171750) and conducted following PRISMA guidelines. Eighteen studies met inclusion/exclusion criteria. Samples comprised 918 participants (340 female) aged 16–38 years. Of these, 603 were regular cannabis users, and 315 were controls.

**Results:**

The literature consistently reported greater brain activity in cannabis users while exposed to cannabis vs neutral stimuli in three key brain areas: the striatum, the prefrontal (anterior cingulate, middle frontal) and the parietal cortex (posterior cingulate/precuneus) and additional brain regions (hippocampus, amygdala, thalamus, occipital cortex). Preliminary correlations emerged between cannabis craving and the function of partially overlapping regions (amygdala, striatum, orbitofrontal cortex ).

**Conclusions:**

Exposure to cannabis-cues may elicit greater brain function and thus trigger cravings in regular cannabis users and thus trigger cannabis craving. Standardised and longitudinal assessments of cannabis use and related problems are required to profile with greater precision the neurobiology of cannabis cue-reactivity, and its role in predicting  cravings and relapse.

**Supplementary Information:**

The online version contains supplementary material available at 10.1007/s00213-021-05973-x.

## Introduction

Cannabis is the most widely used substance globally, with ~ 192 million users in the past year (United Nations Office on Drugs and Crime (UNODC, 2020). A significant and increasing minority of ~ 10% of users consume cannabis on a regular basis (UNODC, 2020). This is concerning as regular cannabis use (i.e. at least once a month; Sutherland et al. 2021) is associated with a range of psychosocial outcomes including severe cannabis use disorders (CUD) and mental health disorders (American Psychological Association (APA), 2013; Hasin et al. 2016) and lower IQ, education and cognitive performance (e.g. working memory; Scott et al. 2018). Cannabis use-related problems are reported to incur a substantial financial burden globally from a range of issues, e.g. traffic accidents, hospital/treatment services, psychological disorders and work absenteeism (UNDOC, 2020). For these reasons, it is critical to understand the pathophysiological mechanisms of regular cannabis use in order to develop effective intervention strategies to prevent these issues and/or mitigate their effects. From a neurobiological perspective, we are yet to fully understand the key processes and brain regions that are associated with regular cannabis use. In spite of this, the implementation of magnetic resonance imaging (MRI) has caused increasingly advanced efforts to identify the pathophysiology of regular cannabis use.

A core feature of regular cannabis use is greater reactivity to cannabis cues vs neutral cues (henceforth called “cue-reactivity” (Jasinska et al. 2014). Greater cannabis cue-reactivity has been robustly demonstrated using self-report (e.g. higher valence, arousal and craving rating) and various psychophysiological indices (e.g. higher heart rate, blood pressure, skin temperature and P300 amplitude (Norberg et al. 2016). Greater cannabis cue-reactivity in regular cannabis users has been posited to develop as a result of repeated cannabis consumption, whereby cannabis-related cues (e.g. paraphernalia, smell, contexts) progressively acquire a rewarding value in that they signal and predate/anticipate the experience of the reward (i.e. pleasure, feeling high) that will come from the consumption of cannabis (Jasinska et al. 2014). Thus, reactivity to cannabis-related cues has been posited to underlie symptoms consistent with a CUD: increased motivation for using cannabis, habitual/repeated cannabis use and in some also the experience of cravings for cannabis (i.e. strong desires, urges and preoccupation to use), loss of control of cannabis use and relapse following attempts to reduce or quit (APA, 2013; Berridge and Robinson 2016; Zilverstand et al. 2018). Notably, cannabis and related products have become increasingly available and advertised (either in a licit or illicit fashion) in outlets online and in communities globally due to trends towards the decriminalisation of recreational and medical cannabis. Therefore, investigating how exposure to cannabis-related cues affect the brain, and how brain alterations in relation to cannabis cue exposure relate to cannabis craving and chronicity of use, is timely to inform users and their relatives in the general community, clinical practitioners and policy-makers (Wilkinson et al. 2014).

Animal studies and meta-analysis of drug cue-reactivity studies (e.g. alcohol, nicotine, cocaine) show that greater reactivity to substance-related cues in regular substance users is ascribed to sensitisation of brain pathways implicated in reward processing with repeated exposure to substances. These include striatal areas implicated in reward processing, limbic regions mediating stress, and prefrontal cortex (PFC) areas implicated in motivation and disinhibition (Koob and Volkow 2016; Noori et al. 2016; Zehra et al. 2018). Specifically, such reward brain pathways would be activated with cannabis consumption in occasional users; however, with repeated cannabis use, the activation of these pathways would occur also in response to exposure to cannabis-related cues that signal/predate cannabis use, thereby triggering repeated/automatic cannabis use behaviour, motivation for using, and in some, also craving and relapse when attempting to cut down or quit (Berridge and Robinson 2016; Zilverstand et al. 2018).

However, the neurobiology of reactivity to cannabis cues in regular cannabis users are yet to be fully mapped. The evidence from functional magnetic resonance imaging (fMRI) studies that have mapped brain function with high-resolution, in-vivo, non-invasively during exposure to cannabis cues in regular cannabis users has yet to be synthetised (Blest-Hopley et al. 2018; Yanes et al. 2018). A careful profiling of the neurobiological correlates of cannabis cue-reactivity is required to further neurobiological theories of addiction (i.e. anticipation/motivation stage) as these are largely based on evidence on substances other than cannabis (Zehra et al. 2018) (Koob and Volkow 2016). A synthesis of the evidence on the neurobiology of cannabis cue-reactivity will also create a knowledge base that can be used to inform the development of neurobiological targets for treatment that aim to mitigate reactivity to cannabis cues, consequent automated use, and in some, craving and relapse.

The first aim of this systematic review is to synthesise the evidence to date on the brain functional correlates of cannabis cue-reactivity in regular cannabis users examined using fMRI tasks which entail participants’ exposure to cannabis *vs* neutral stimuli (henceforth CAN vs NEU). The secondary aim of this review is to summarise the evidence on the associations between brain function in cannabis users (while exposed to CAN vs NEU stimuli) and the level of various variables including subjective cannabis craving, cannabis exposure (e.g. duration, dosage, frequency), cannabis use-related problems and exposure to substances other than cannabis. An additional aim is to overview the methodologies used to measure cannabis cue-reactivity using fMRI in regular cannabis users in order to inform on the methodological standards in this area of research.

## Method

### Search strategy

This review was pre-registered via PROSPERO (ID: CRD42020171750). A systematic search of the literature to date (5 November, 2020) was reported in-line with the Preferred Reporting Items for Systematic Reviews and Meta-Analysis (PRISMA) guidelines (Moher et al. 2009), full checklist in Online Resource 1. Searches were completed using PsycINFO, PubMed and Scopus databases. Search terms included (“cannabis use disorder” OR cannabis OR marijuana) AND (fMRI OR “functional magnetic resonance imaging” OR MRI OR “magnetic resonance imaging” OR “brain activity” OR “brain function” OR connectivity OR “neural activity”) AND (“cue-reactivity” OR “cue-salience” OR craving OR reward OR sensitization). All terms were searched within title, abstract and keywords. No time limits were placed on the search.

### Inclusion and exclusion criteria

Inclusion criteria were as follows: (i) the manuscript was written in English; (ii) the sample included human participants; (iii) the mean age of the sample ranged between 14 and 65 years; (iv) the sample comprised people who regularly use cannabis (i.e. at least once a month; Sutherland et al. 2021) or meeting criteria for a cannabis use disorder/dependence); (v) fMRI was used as a technique to measure brain function; vi) a cue-reactivity fMRI task was used to measure brain function; (vii) brain function was measured via contrasting presentation of CAN vs NEU stimuli; and (viii) the manuscript was published in peer-reviewed journal.

Exclusion criteria were as follows: (i) the sample was defined as endorsing a diagnosis of any major mental health disorder (e.g. depression, schizophrenia) or neurological disorders (e.g. epilepsy); (ii) the sample had regular/disordered/dependent use of substances other than cannabis, alcohol or tobacco (as defined by each study); (iii) brain integrity was measured using neuroimaging techniques other than fMRI (e.g. structural MRI, diffusion-weighted imaging, electroencephalography, positron emission tomography); (iv) the study was not an experiment (e.g. single case report, case studies, review or meta-analysis); and (v) the manuscript was not published in a peer-reviewed journal (e.g. conference abstract, book chapter, dissertation).

**Fig. 1:**
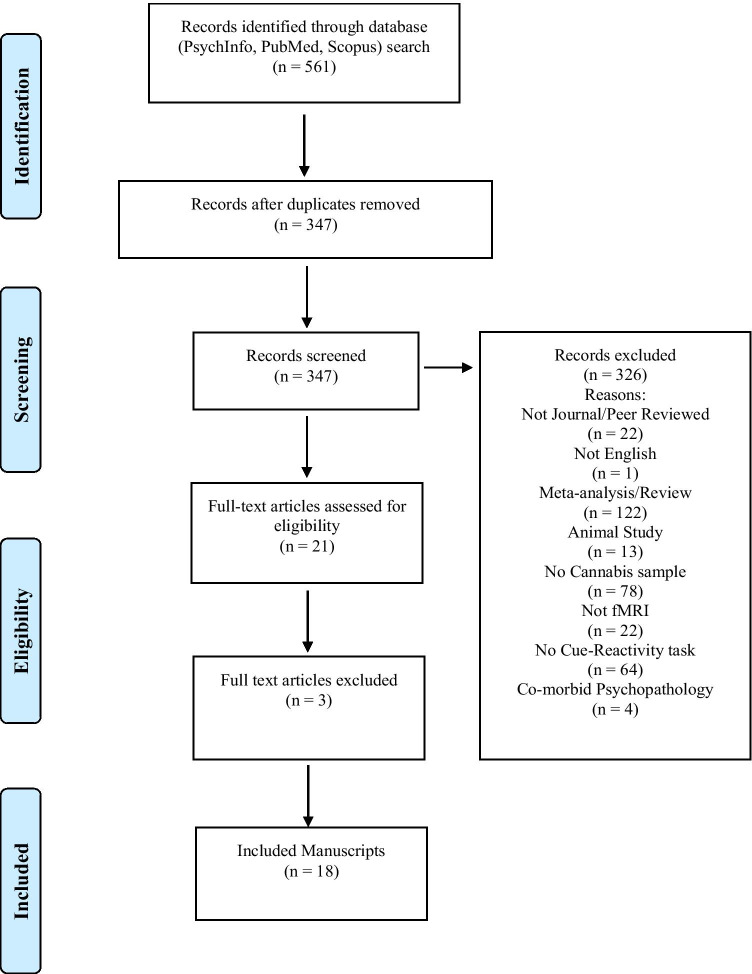
PRISMA flowchart for study selection process (based on Moher et al. 2009)

Figure 1 outlines the PRISMA flowchart which summarises the systematic study selection process for inclusion in this review. Screening of all records’ titles, abstracts and full-texts (after duplicates were removed) against the inclusion and exclusion criteria was done independently by two student researchers (H.S., H.T.). The resulting article selection was then disclosed, and any discrepancies were resolved via discussion with a senior researcher (V.L.). As a result of this process, 18 manuscripts were identified as eligible for this review.

### Data extraction

The following data was extracted from all studies: (i) study characteristics (e.g. first author, year of publication and recruitment strategy); (ii) sample socio-demographic characteristics (e.g. sample size, age, sex); (iii) level of cannabis use and related problems in the cannabis group (e.g. dosage, duration, age of onset, frequency/occasions, abstinence duration); (iv) method used to analyse fMRI data (e.g. whole brain, ROI); (v) brain functional differences comparing exposure to CAN vs NEU stimuli (a) within cannabis users and (b) between cannabis users compared to controls (i.e. additional brain functional differences between cannabis subgroups were extracted); and (vi) correlations between brain function (while exposed to CAN vs NEU stimuli) and behavioural variables (e.g. subjective craving, level of cannabis use).

Supplementary materials (including Supplementary Tables 1–4) available in Online Resource 1 overview data that was additionally extracted: (i) inclusion/exclusion criteria and assessment methods at study level (e.g. comorbid psychopathology, concurrent substance use, medical conditions); (ii) group inclusion/exclusion criteria and assessment methods at study level (e.g. severity and/or diagnosis of cannabis use disorder/dependence, treatment status and abstinence duration in the cannabis group, level of alcohol and tobacco use); (iii) biological measures of cannabinoids from various specimens; (iv) details of the cannabis cue-reactivity fMRI task (e.g. craving rating, stimuli type, presentation protocol); and (v) technical characteristics of imaging data acquisition (e.g. fMRI acquisition parameters, MRI scanner strength and manufacturer, number of head coil’ channels).

### Additional data handling

We extracted data from cross-sectional comparisons of brain function within cannabis users (CAN vs NEU) and between cannabis users and controls. The design of two studies was prospective with assessment of cannabis users and controls at baseline (Cousijn et al. 2013) and 3 years later (Vingerhoets et al. 2016). From these datasets, results on the primary outcome variable (i.e. brain function comparing cannabis vs control groups) were extracted during baseline administered of the cue-reactivity fMRI task (Cousijn et al. 2013) and associations with cannabis use patterns and related problems 3 years later (Vingerhoets et al. 2016).

### Risk of bias

Results from the quality assessment showed consistency in the quality of studies included in this review; see Supplementary Table 5. All 18 studies stated the research question/s clearly, with the study population/s specifically defined and selected from similar populations and time periods. Inclusion/exclusion criteria were applied uniformly to all participants across all included studies. The independent and outcome variables were prespecified and implemented consistently across all studies, with sufficient time so that one could expect to see an association between exposure and outcome if it existed. No study provided a sample size justification, power description or effect estimates. Similarly, no study blinded the researchers to the group status of the participants. Only eight of the 18 studies controlled for potential confounding variables in their statistical analysis, which were also inconsistent. Only four studies respectively controlled for cannabis problems/dependence severity, cannabis use patterns and age. Five studies controlled for alcohol, and three controlled for cigarettes, two for IQ/education years and one for lifetime use of other psychotropic substances.

## Results

A total of 18 studies were included in this review (Bitter et al. 2014; Charboneau et al. 2013; Cousijn et al. 2013; de Sousa Fernandes Perna et al. 2017; Feldstein Ewing and Chung 2013; Filbey and Dunlop 2014; Filbey et al. 2016; Filbey et al. 2009; Goldman et al. 2013; Karoly et al. 2019; Kleinhans et al. 2020; Kuhns et al. 2020; Vingerhoets et al. 2016; Wetherill et al. 2014; Wetherill et al. 2016; Wetherill et al. 2015; Yoo et al. 2020; Zhou et al. 2019).

### Overview of groups compared

We extracted brain function during exposure to CAN vs NEU stimuli, in the following groups: (i) within cannabis users in 12 out of 18 studies (Charboneau et al. 2013; Cousijn et al. 2013; de Sousa Fernandes Perna et al. 2017; Feldstein Ewing et al. 2013; Filbey et al. 2009; Filbey and Dunlop 2014; Filbey et al. 2016; Goldman et al. 2013; Karoly et al. 2019; Kleinhans et al. 2020; Wetherill et al. 2014; Wetherill et al. 2015) and (ii) between cannabis users and non-using controls in eight studies (Bitter et al. 2014; Cousijn et al. 2013; de Sousa Fernandes Perna et al. 2017; Filbey et al. 2016; Kleinhans et al. 2020; Kuhns et al. 2020; Yoo et al. 2020; Zhou et al. 2019). We additionally extracted brain function during exposure to CAN vs NEU stimuli between distinct cannabis subgroups, which was reported in 1–2 studies: dependent vs non-dependent users (2 studies) (Filbey and Dunlop 2014; Zhou et al. 2019); high vs low problem cannabis use (2 studies) (Cousijn et al. 2013; Vingerhoets et al. 2016); early vs late cannabis use onset (1 study) (Cousijn et al. 2013; Wetherill et al. 2016, frequent vs sporadic (*n* = 1); cannabis use only vs cannabis *and* tobacco use (Kuhns et al. 2020); and male vs female cannabis users (1 study) (Wetherill et al. 2015).

### Overview of sample socio-demographic and cannabis use characteristics

Table 1 overviews the socio-demographic characteristics of the reviewed samples. The reviewed samples comprised a total of 918 participants (340 female), of which 603 were cannabis users and 315 were non-cannabis using controls (i.e. henceforth controls). The sample size ranged from 12 to 144 participants aged between 16 and 38 years. Males were slightly represented in ten studies, and the ratio of males and females was even in the remainder eight studies. Participants’ recruitment source was described in all studies but one (de Sousa Fernandes Perna et al. 2017). Most samples were recruited from the general community (15 studies, using flyers, newspapers, internet, media), and two samples were recruited from other sources, e.g. juvenile justice programs (Feldstein Ewing et al. 2013) and drug counselling services (Zhou et al. 2019).

**Table 1:** Sample socio-demographics characteristics and cannabis use levels

Author, year	Total *N* (female)			Age, *yrs* mean (SD)		Education, *yrs* mean (SD)		Cannabis use level				
	Cannabis Subgroups	Cannabis	Control	Cannabis	Control	Cannabis	Control	Age onset, *yrs*	Duration, *yrs*	Frequency, *day/occasions per week*	Weekly dosage, *grams/joints*	Abstinence duration (hrs)
Filbey, 2009	_	31 (3)	_	23.7 (7.3)	_	_	_	17	7	6 day/wk, 21 occ/wk	_	_
Charboneau, 2013	_	16 (11)	_	23.7 (3.9)	_	_	_	15	_	16 occ/wk	_	14
Cousijn, 2013	Frequent	31 (11)	21 (8)	21.3 (2.3)	22.1 (2.5)	_	_	_	3	5 day/wk	3 g/wk, 10 joints/wk^c^	_
	Sporadic	20 (7)		22.1 (2.4)		_	_	_	_	_	10 joints/life^c^	_
	High problem	16 (7)		21.4 (2.4)		_	_	-	3	5 day/wk	4 g/wk, 12 joints/wk^c^	_
	Low problem	15 (4)		21.1 (2.3)		_	_	_	2	5 day/wk	2 g/wk, 12 joints/wk^c^	_
Goldman, 2013	_	12 (2)	_	37.6 (10.7)	_	13.0 (2.0)	_	_	19	7 day/wk^c^	21 g/wk^c^ 84 joints/wk^c^	_
Feldstein Ewing, 2013	_	43 (7)	_	16.1 (1.1)	_	_	_	12 ^a^	_	4 day/wk^c^	_	_
Bitter, 2014	_	13 (5)	15 (9)	19.0 (1.0)	18 (2)	_	_	_	_	_	2 g/wk^c^ 8 joints/wk^c^	26
Filbey, 2014	Dependent	37 (9)	_	24.5 (6.9)	_	13.8 (3.2)	_	18	6	6 day/wk^c^	_	_
	Non-dependent	34 (9)		24.5 (8.1)		13.4 (2.2)		17	8	6 day/wk^c^		
Wetherill, 2014	_	20 (12)	_	29.1 (9.7)	_	12.4 (3.0)	_	_	11	7 day/wk^c^	14 g/wk^c^	_
Wetherill, 2015	Female	17	_	30.0 (6.8)	_	13.0 (2.2)	_	_	11	6 day/wk	21 g/wk^c^	_
	Male	27		29.3 (8.2)		13.0 (1.5)					28 g/wk^c^	
Filbey, 2016	_	53 (20)	68 (35)	30.7 (7.5)	31.4 (10.2)	13.1 (3.1)	16.8 (2.8)	_	12	_	14 g/wk^c^ 2 THC/CR (ng/ml)^b^	_
de Sousa Fernandes Perna, 2017	_	21 (6)	20 (10)	22.5 (2.3)	22.5 (2.3)	_	_	_	_	5 occ/wk^c^	1.2 THC (µ /l) 0.4 THC-Oh (µ /l) 16 THC-COOH (µ /l)	_
Vingerhoets, 2016	Baseline	12 (4)	_	20.9 (2.4)	_	13.8 (2.2)	_	_	_	5 day/wk	3 g/wk	_
	Follow-up	11 (3)	_	24.1 (2.4)	_	16.7 (3.2)	_	_	_	5 day/wk	3 g/wk	_
Wetherill, 2016	Early onset	15 (8)	_	29.0 (2.0)	_	12.5 (0.4)	_	13	5	_	21 g/wk^c^	_
	Late onset	26 (7)		28.7 (1.4)		13.1 (0.3)		20			28 g/wk^c^	
Karoly, 2019	_	40 (21)	_	18.8 (1.0)	_	_	_	_	_	5 day/wk^c^	_	_
Zhou, 2019	Dependent	18 (0)	44 (0)	22.9 (2.7)	23.2 (4.3)	15.3 (2.5)	15.6 (2.5)	15	5	_	6 g/wk^c^	40
	Non-dependent	20 (0)		21.5 (2.5)			14.4 (1.7)	16			4 g/wk^c^	83
Kuhns, 2020	Cannabis + Tobacco	16 (6)	14 (8)	21.3 (2.0)	20.7 (2.5)	_	_	_	_	5 day/wk^c^	3 g/wk^c^	_
	Cannabis only	18 (10)	18 (10)	20.7 (2.1)	21.6 (2.4)	_	_	_	_	4 day/wk^c^	3 g/wk^c^	
Yoo, 2020	_	54 (23)	90 (45)	29.4 (7.9)	29.4 (10.2)	_	_	19	10	_	14 g/wk^c^ 2 THC/creatinine (ng/ml)	79.4
Kleinhans, 2020	_	25 (12)	25 (12)	26.2 (4.2)	26.2 (5.1)	_	_	17	4	2–4 day/wk^d^	3 g/wk^c^	98.4

*Note: Yrs* years, *N* number, *SD* standard deviation, *wk* week, *occ* occasions, *Edu* education, *THC* tetrahydrocannabinol, *THC-Oh* 11-Hydroxy-Δ^9^-tetrahydrocannabinol, *THC-COOH* 11-Nor-9-carboxy-Δ^9^-tetrahydrocannabinol, *ADHD* attention deficit hyperactivity disorder

^a^Age of first use

^b^Measured during pre-assessment abstinence period

^c^Estimated averages calculated via past number of day per month divided into weeks and number of grams per day or lifetime into weeks to aid comparability across studies

^d^80% of users

### Overview of cannabis use levels

Table 1 overviews the levels of cannabis use in the cannabis groups. The age of onset of cannabis use ranged from 12 to 20 years, with a mean age of onset of 16 years across studies. The duration of cannabis use varied widely from 2 to 19 years, with a mean duration of cannabis use of 8 years across studies. Most studies reported how often cannabis was currently used in either weekly consumption days (11 studies) or occasions (3 studies). The level of cannabis use was from 5 days to everyday of the week, and the number of weekly cannabis use occasions ranged from 5 to 21. Cannabis dosage was measured in 13 of the 18 studies, using heterogeneous metrics and over different period of times. These ranged from 2 to 28 grams a week (11 studies) and 8–84 joints a week (2 studies). Only two studies quantified THC metabolites in urine, which corroborated presence of cannabis use. The duration of abstinence from cannabis at the time of scan was reported only by four studies and ranged widely from 14 hours to ~ 4 days.

### Overview of fMRI methods

Table 2 summarises the fMRI data analysis methods used. The most consistently used fMRI data analysis method was a region of interest approach (ROI; 7 studies), followed by a whole brain approach (5 studies) and by a seed-based connectivity approach (seed/network to whole brain; 3 studies). Some of these studies used multiple methods concurrently: ROI *and* whole brain (5 studies;Cousijn et al. 2013; Goldman et al. 2013; Karoly et al. 2019; Kleinhans et al. 2020; Kuhns et al. 2020; Zhou et al. 2019).

**Table 2:** Summary of fMRI analysis method and brain alteration during cue-reactivity task (CAN > NEU) in cannabis users

Author, year	fMRI data analysis approach	Regions of interest	Brain functional differences using contrast CAN > NEU		
			Within cannabis users	Cannabis users vs control**s**	Cannabis subgroups
Filbey, 2009	Whole brain	_	Thalamus, amygdala, frontal (inferior, middle), lOFC, pre/postcentral, dACC, parietal (inferior), precuneus, temporal (superior, middle), fusiform, insula, occipital (middle, inferior), cerebellum, VTA	_	_
Charboneau, 2013	Whole brain	_	Amygdala, hippocampus, uncus, temporal (inferior, middle), fusiform, occipital (inferior middle), lingual, cerebellum	_	_
Cousijn, 2013^a^	Whole brain	_	PCC, precuneus, frontal (medial, superior), OFC, occipital/angular (lateral)		*High* > *low-problem*: frontal (medial), temporal (pole) *Frequent* + *Tobacco* = *Frequent Non-tobacco*^*b*^
	ROI	Amygdala, OFC, ACC, striatum/VTA		*CB* > *HC:* frontal (medial), temporal pole, VTA	*High* > *low-problem*: striatum (NAcc, caudate, putamen), ACC, OFC
Feldstein Ewing, 2013	Whole brain	_	Thalamus, striatum (caudate), parahippocampus, uncus, frontal (superior, middle, inferior), pre/postcentral, ACC, parietal (inferior, superior), temporal (inferior, superior), insula, cerebellum (tonsil), occipital (middle)	_	_
Goldman, 2013	ROI	Amygdala, hippocampus, OFC, striatum (ventral), insula (ventral)	Amygdala, hippocampus	_	_
Bitter, 2014	ROI	Amygdala, thalamus, striatum (NAcc), vmPFC	*CAN* = *NEU*	*CB* > *HC:* striatum/NAcc	_
Filbey, 2014	Seed-based	Amygdala, hippocampus, OFC, ACC, striatum (Nacc, VTA), insula	Striatum (NAcc) – striatum (caudate), ACC, cerebellum	_	*Dependent* > *non-dependent:* amygdala –frontal (middle, inferior), temporal (superior); ACC – postcentral, parietal (superior, inferior), precuneus *Dependent* < *non-dependent:* NAcc – postcentral, parietal (superior, inferior), OFC – frontal (superior), pre/postcentral; hippocampus – precuneus
Wetherill, 2014	ROI	Amygdala, hippocampus, mOFC, periACC, striatum (ventral), insula	Amygdala, striatum (ventral), insula	_	_
Wetherill, 2015	ROI	Amygdala, hippocampus, mOFC, periACC, striatum (ventral), insula	Hippocampus, amygdala, lOFC, ACC, striatum, insula	_	*Male* = *female*
Filbey, 2016	Whole brain	_	Parahippocampus, thalamus, frontal (medial, inferior), ACC, precuneus, cerebellum, cuneus, occipital (lateral) *CAN* < *NEU:* occipital (lateral), cuneus, precuneus	*CB* > *HC:* caudate, ACC, precuneus, PCC, subcallosal, cerebellum	_
de Sousa Fernandes Perna, 2017	ROI	Striatum (putamen, caudate, pallidum)	pallidum	*CB* = *HC*	_
Wetherill, 2016	ROI	Amygdala, hippocampus, mOFC, periACC, striatum (ventral), insula	_	_	*Early* > *late onset:* striatum (dorsal) *Early* < *late onset*: striatum (ventral)
Karoly, 2019	Whole brain	_	Parahippocampus, thalamus, frontal (superior, medial), fusiform, PCC, cuneus, parietal (inferior), precuneus, temporal (inferior), occipital (middle)	_	_
Zhou, 2019	Whole brain	_	_	*CB* > *HC:* striatum (NAcc, ventral caudate), frontal (inferior, medial, superior), mPFC, ACC, parietal (inferior, superior), PCC, precuneus, temporal, fusiform, occipital	*Dependent* > *non-dependent:* PCC, precuneus
	ROI	Striatum (ventral, dorsal)	_	_	*Dependent* > *non-dependent:* striatum (dorsal)
	Seed-based	Striatum (ventral, dorsal)	_	*CB dependent* > *HC*: striatum (dorsal) – frontal (inferior), vACC *CB dependent* < *HC:* striatum (ventral/dorsal) – hippocampus, amygdala	_
Kuhns, 2020	Whole brain	_	_	*HC* + *tobacco* > *CB* + *&—tobacco*: frontal pole, inferior frontal	
	ROI	Amygdala, OFC, ACC, striatum/VTA	**_**	*CB-tobacco* > *HC-tobacco:* amygdala *HC* + *Tobacco* > *CB* + *tobacco:* striatum, amygdala, ACC	*CB* + *Tobacco* = *CB no tobacco*
Yoo, 2020	ROI	347 regions: DMN, CEN, SN, amygdala, NAcc	_	*CB* > *HC:* NAcc – central executive network	
Kleinhans, 2020	ROI	Striatum (NAcc, pallidum, VTA)	_	*CB* = *HC*	_
	Whole brain	_	*Cannabis* > *flower (picture and odour):* parahippocampus, amygdala, striatum (NAcc, putamen), frontal (superior, middle, medial, inferior, pole), OFC, para/subcallosal/ACC, juxtapositional, precentral, temporal (middle, inferior, superior), fusiform, temporal pole, intracalcarine, cuneal, angular, occipital (pole), crus, lingual *Cannabis* > *flower (picture):* parahippocampus, amygdala, striatum (pallidum), OFC, lingual, temporal (inferior, middle), intracalcarine, temporal, occipital (pole), fusiform, crus, lingual *Cannabis* > *flower (odour):* striatum (putamen), central (pre, post), ACC, supramarginal, angular, frontal (pole, operculum), juxtapositional, parietal (operculum, superior), temporal (middle, planum temporale), occipital (lateral), cerebellum (crus)	*Cannabis* > *flower (picture and odour):* parietal (superior, precuneus), occipital cortex (lateral superior), angular	_

Note: *ROI* region of interest, *OFC* orbitofrontal cortex, *VTA* ventral tegmental area, *ACC* anterior cingulate cortex, *PCC* posterior cingulate cortex, *PFC* prefrontal cortex, *NAcc* nucleus accumbens, *CB* cannabis, *DMN* default mode network, *CEN* central executive network, *SN* salience network

^a^Baseline data forVingerhoets et al. (2016)

^b^Subgroup demographics not reported

### Summary of ROIs examined across the studies

A total of 347 ROIs were examined across the 18 studies. The most examined ROI was the ventral striatum/nucleus accumbens (NAcc; 13 studies) followed by the amygdala (9 studies). These were followed by the orbitofrontal cortex (OFC) and the dorsal striatum (7 studies, respectively; the latter included the caudate, 2 studies; pallidum, 2 studies; and the putamen, 1 study) and then by the anterior cingulate cortex (ACC; 6 studies) and the insula and the hippocampus (5 studies, respectively). Single studies examined the thalamus and a variety of other regions, as well as networks (i.e. default mode, salience, central executive). Table 2 overviews results on differences in brain function between exposure to CAN vs NEU within cannabis users, between cannabis users and controls and between various cannabis using subgroups.

### Brain functional differences in cannabis users, during exposure to CAN vs NEU stimuli

Greater brain activity was reported in 11 of 12 within cannabis using samples while exposed to CAN vs NEU stimuli. Single studies reported lower activity (i.e. in parietal and occipital cortices) and non-significantly different brain function, while cannabis users were exposed to CAN vs NEU stimuli. Greater brain function in cannabis users while exposed to CAN vs NEU stimuli was located most consistently in the hippocampus/parahippocampus (8 studies) and the amygdala (6 studies), followed by the thalamus, the PFC (middle frontal gyrus, ACC), parietal regions (precuneus, posterior cingulate cortex (PCC), inferior gyrus), the occipital cortex (5 studies each respectively) and other PFC regions (OFC, inferior/superior frontal gyrus), striatum (ventral), insula, fusiform/inferior temporal gyri and cerebellum (4 studies). The activity of additional regions was reported to be greater in cannabis users while exposed to CAN vs NEU by three studies (for each region): precentral/postcentral gyrus; temporal gyrus (inferior, middle, superior) and inferior occipital gyrus. Greater function (CAN vs NEU) in cannabis users was reported by ≤ 2 studies in other striatal, parietal and occipital areas. A single study reported higher functional connectivity during exposure to CAN vs NEU stimuli between the NAcc and the caudate head, ACC and cerebellum.

### Brain functional differences between cannabis users and controls, during exposure to CAN vs NEU stimuli

Greater brain activity was found in cannabis users compared to controls while exposed to CAN vs NEU stimuli in most studies that compared these groups (7 out of 8 studies). The location of greater activity was most consistently in the striatum (NAcc, caudate, 3 studies respectively) and parietal cortex (precuneus). Additional regions with greater activity were reported by two studies, respectively, and included the PFC (ACC, middle frontal gyrus) and parietal regions (superior parietal cortex, PCC).

Non-significant differences in brain function between cannabis users and controls emerged in two studies. Single studies reported greater brain function in additional regions and lower brain function in cannabis users compared to controls while exposed to CAN vs NEU stimuli: lower activity in the striatum, amygdala and other areas (e.g. inferior frontal gyrus, ACC, amygdala) and lower functional connectivity between the striatum and the hippocampus/amygdala.

### Brain functional differences between cannabis user subgroups

A range of findings emerged from three or less studies that compared varying cannabis using subgroups. Greater function of striatal regions (caudate, putamen, pallidum, NAcc) was reported in more severely using cannabis subgroups: dependent vs non-dependent users, high vs low problem users, frequent users vs sporadic users and early onset vs late onset users, whereas greater function in the ventral striatum was reported in non-dependent vs dependent and connectivity between NAcc—parietal/postcentral gyri in late vs early onset cannabis users.

Greater and lower function of parietal regions was also reported in dependent cannabis users by two studies. These included (i) greater activity of the precuneus and the PCC in dependent vs non-dependent cannabis users and (ii) within dependent cannabis users, greater/lower functional connectivity between parietal areas (postcentral gyrus, superior and inferior gyri) and other regions (ACC, NAcc) and greater functional connectivity between other parietal regions (precuneus) and the ACC. Greater function of PFC regions was reported in two studies comparing high vs low problem users (higher activity of the medial frontal gyrus, ACC, OFC) and within non-dependent cannabis users (greater functional connectivity between the OFC and the superior frontal, precentral and postcentral gyri). In a single study, greater amygdala connectivity with the PFC (middle, inferior) and temporal gyrus (superior) was reported in dependent cannabis users, and greater hippocampus connectivity with the precuneus was reported within non-dependent cannabis users.

No difference in brain function was reported between other cannabis user subsamples, such aswith vs without tobacco use or male vs female.

### Overview of associations between brain function (CAN vs NEU stimuli) and other variables

Table 3 overviews results from studies (all but two) that examined the association between the level of brain function and that of various variables. The results are overviewed below grouped by the type of variable that was correlated with brain function in the following order: subjective craving, cannabis exposure, level of cannabis use-related problems and use of substances other than cannabis.

**Table 3:** Associations between brain function during cue-reactivity tasks and cannabis craving, dependence, level of use and substance use level

Author, year	fMRI data analysis approach	Cannabis cravings/urges/arousal	Cannabis use/dependence level	Substance use level
Filbey, 2009	Whole brain	_	( +) *Marijuana Problem Scale* and mOFC, ACC, striatum (NAcc)	_
Charboneau, 2013	Whole brain	(NS) MCQ	_	_
Cousijn, 2013	Whole brain, ROI: amygdala, OFC, ACC, striatum/VTA	*Frequent users:* (-) MCQ and striatum (putamen), DLPFC	(NS) grams/week, joints/lifetime, duration	(NS) Nicotine use duration, cigs/day, FTND, presence vs absence of tobacco use
Feldstein Ewing, 2013	Whole brain	(NS) VAS	_	_
Goldman, 2013	ROI: amygdala, hippocampus, OFC, striatum (ventral), insula (ventral)	( +) MCQ and striatum (ventral), OFC (medial, lateral)	_	_
Bitter, 2014	ROI: amygdala, thalamus, striatum (NAcc), vmPFC	(NS) MCQ	(NS) joints/mo	_
Filbey, 2014	Seed-based: amygdala, hippocampus, OFC, ACC, striatum (Nacc, VTA), insula	( +) MCQ and connectivity in striatum (NAcc) – thalamus, pulvinar, cerebellum ( +) MCQ and connectivity in amygdala – precentral, frontal (middle)	_	_
Wetherill, 2014	ROI: amygdala, hippocampus, mOFC, periACC, striatum (ventral), insula	_	_	_
Wetherill, 2015	ROI: amygdala, hippocampus, mOFC, periACC, striatum (ventral), insula	(NS) MCQ *Female:* ( +) MCQ and insula (anterior) (-) MCQ and OFC (lateral) *Male:* ( +) MCQ and striatum	_	_
Filbey, 2016	Whole brain	( +) VAS and striatum (caudate, globus pallidus), OFC, amygdala, ACC, thalamus, parahippocampus, frontal (inferior, middle), PCC, cerebellum, temporal (superior), insula ( +) MCQ and insula, pre/postcentral, parietal (inferior), cuneus, temporal (superior)	(NS) N of DSM-IV cannabis dependence symptoms ( +) urine THC (ng/ml) and lingual gyrus, cuneus	_
de Sousa Fernandes Perna, 2017	ROI: striatum (putamen, caudate, pallidum)	_	_	_
Vingerhoets, 2016	ROI: amygdala, OFC, ACC, striatum/VTA	_	( +) CUDIT (cannabis problems) and striatum (putamen), VTA (NS) CB use (grams/weekly)	_
Wetherill, 2016	ROI: amygdala, hippocampus, mOFC, periACC, striatum, insula	*Early onset:* ( +) MCQ and striatum (dorsal) *Late onset:* (NS) MCQ	_	_
Karoly, 2019	ROI: amygdala, OFC, striatum (ventral)	(NS) MCQ	(NS) days/lifetime, episodes/day	(NS) AUDIT (alcohol use)
Zhou, 2019	ROI: striatum (ventral, dorsal)	*Dependent:* ( +) VAS and striatum (dorsal/ventral caudate) *Non-dependent:* (-) VAS and striatum (dorsal/ventral caudate)	_	_
Kuhns, 2020	Whole brain/ROI: Amygdala, OFC, ACC, striatum/VTA	_	*Cannabis* + *tobacco users:* cannabis grams/wk and VTA^a^ *Cannabis* + *non-tobacco users:* (NS) cannabis grams/wk	(NS) Group × tobacco use interaction
Yoo, 2020	ROI: 347 regions, DMN, CEN, SN, amygdala, NAcc	( +) VAS and connectivity between striatum (NAcc)—networks (SN, DMN, CEN) ( +) MCQ and connectivity within central executive network ( +) MCQ and modularity of whole brain	_	_
Kleinhans, 2020	Whole brain, ROI: striatum (NAcc, pallidum, VTA)	*Cannabis* > *Flower:* ( +) VAS and striatum (caudate, putamen, pallidum), OFC, amygdala, insula, para/ACC, post/precentral, opercular (central), Heschl’s, planum polare, temporal pole, parietal (operculum), temporal (planum temporale), frontal (inferior), lingual/cuneal, calcarine (intra, supra), precuneus, occipital (lateral), juxtapositional *All other contrasts:* (NS) VAS	_	_

Note: *ROI* region of interest, *NS* not significant, *OFC* orbitofrontal cortex, *VTA* ventral tegmental area, *ACC* anterior cingulate cortex, *PCC* posterior cingulate cortex, *PFC* prefrontal cortex, *NAcc* nucleus accumbens, *SN* salience network, *CEN* central executive network, *DMN* default mode network, *MCQ* Marijuana Craving Questionnaire, *VAS* visual analogue scale, *CUDIT* Cannabis Use Disorders Identification Test, *AUDIT* Alcohol Use Disorders Identification Test, *FTND* Fagerstrom Test for Nicotine Dependence.

^a^Direction of association not specified

### Brain function and subjective cannabis craving

Overall, 13 studies examined the association between subjective cannabis craving and brain activity. Of these, correlations were run with brain function measured (i) in specific ROIs (9 studies) or across the whole brain (4 studies) (ii) as either activity (11 studies) or connectivity (2 studies).

#### Correlations with ROI

The nine studies that focused on ROIs ran a total of 51 correlations between brain function and subjective craving. The results were non-significant in about two-third of these correlations (*n* = 38) and significant in opposite directions in the remainder correlations (positive in 8 studies and negative in 3 studies).

#### Correlations with regions from whole-brain approach

Of the four studies that used a whole brain approach, only two found significant correlations and reported 36 consistently positive correlations between a range of brain areas and subjective craving.

#### Direction and location of correlations with subjective craving

The direction of the significant correlations between subjective craving and brain function was mixed for some of the examined regions (i.e. both positive and negative correlations were reported). The most consistently reported region that was correlated with subjective craving was the dorsal striatum (i.e. putamen, pallidum, caudate; 6 studies), followed by the OFC (4 studies), the amygdala and the insula (3 studies, respectively). Other regions were reported to be significantly correlated with subjective craving by two studies (i.e. ventral striatum), the inferior frontal gyrus and pre/post central gyri, or single studies (i.e. PFC, parietal, temporal, limbic areas and cerebellum).

The direction of correlations between subjective craving and striatal (dorsal) activity was mixed, with a total of four positive correlations across four subgroup:(i) within cannabis users, (ii) in male cannabis users, (iii) early onset users and (iv) dependent users, and two negative correlations in two subgroups: (i) within cannabis users and (ii) non-dependent users. In the OFC, three positive correlations were within cannabis users, and one negative correlation was reported in female users. In the ventral striatum, one positive correlation was within cannabis users, and one negative correlation was reported in male users. Correlations reported in single studies were all positive, with the exception of a single negative correlation with the dlPFC in frequent cannabis users.

#### Correlations with functional connectivity

Two studies reported significant correlations between subjective craving and functional connectivity between two key regions (i.e. NAcc, amygdala) and the function/modularity of other regions/networks.

### Brain function and levels of cannabis exposure

Six studies examined the association between brain function and the level of cannabis use (frequency/quantity) and found non-significant results. There were two exceptions to this: single studies found significant correlations between brain function in distinct regions (VTA, lingual gyrus/cuneus) and cannabis dosage, i.e. weekly cannabis gram consumption in cannabis + tobacco users and greater urinary THC/creatinine metabolites (THC-COOH ng/ml), respectively.

### Brain function and level of cannabis use related problems

Three studies examined the association between brain function and level of cannabis use-related problems. Two of these reported positive correlations between the activity of the PFC (mOFC, ACC) and the striatum/NAcc and greater Marijuana Problem Scale scores and between the activity of the dorsal striatum/putamen and Cannabis Use Disorders Identification Test scores at baseline and 3 years later. There was no association between brain function and the severity of DSM-IV cannabis dependence symptoms.

### Brain function and level of use of substances other than cannabis

Two studies examined associations between brain function and level of cigarette use in cannabis users and reported non-significant results (i.e. number of cigarettes/day, duration of use, Fagerstrom Test of Nicotine Dependence scores and cannabis users with vs without concurrent tobacco use). A single study found non-significant associations between brain function and Alcohol Use Disorders Identification Test scores.

## Discussion

To our knowledge, this is the first systematic review of the literature to date on the functional neural correlates of cue-reactivity fMRI tasks, while regular cannabis users are exposed to cannabis vs neutral stimuli (i.e. CAN vs NEU). The literature consistently reported greater brain activity in cannabis users in three key brain areas: the striatum, the PFC (ACC, middle frontal) and the parietal cortex (PCC/precuneus; relative to controls) and additional brain regions (e.g. hippocampus, amygdala, thalamus, occipital cortex) among cannabis users in studies without controls. Early evidence showed associations between greater brain function in similar brain regions (e.g. dorsal striatum, OFC, amygdala, insula) during cannabis cue-reactivity and higher subjective cannabis craving. The methodologies used to assess cannabis users and cue-reactivity using fMRI tasks varied widely between studies. Overall, the evidence points to greater brain function during cannabis cue-reactivity in regular cannabis users, and such greater brain function may be associated with stronger cannabis craving in response to exposure to cannabis-related cues and to a need for improved standardised assessment of the neurobiology of cue-reactivity in cannabis users.

The literature to date shows that reactivity to CAN vs NEU cues is consistently associated with greater brain function in addiction relevant pathways encompassing the striatum, the PFC and parietal regions implicated in cognitive processes reportedly different between cannabis users vs controls (i.e. reward processing, motivation/disinhibition and cognitive control; Blest-Hopley et al. 2018; Yanes et al. 2018). The results from the literature on cue-reactivity in regular cannabis users are consistent with other existing findings from samples of cannabis and other substance users. First, the location of these functional differences is consistent with that reported by meta-analyses of fMRI studies in cannabis users while performing a variety of cognitive tasks (e.g. attention, memory, inhibition, reward processing; Blest-Hopley et al. 2018; Yanes et al. 2018). Thus, altered brain function in cannabis users might occur across a variety of cognitive tasks including but not limited to cannabis cue-reactivity. Second, the location of greater activity in cue-reactivity tasks (i.e. striatum, PFC, parietal regions) overlapped with that reported in cannabis users during reward processing fMRI tasks other than cue-reactivity (e.g. gambling; Yanes et al. 2018) but not distinct during cognitive control and attention-related tasks (e.g. Go/No-Go, N-back; Yanes et al. 2018). Therefore, alteration of specific pathways might be ascribed with altered reward processing in regular cannabis users. Third, the location of the group differences reported in this review (e.g. dorsal striatum, ACC, middle frontal gyrus, PCC/precuneus and temporal regions) was consistent with that reported in meta-analyses of brain function measured with fMRI tasks of cue-reactivity predominantly to substances other than cannabis (Noori et al. 2018). Thus, reactivity to any substance-related cues might recruit a common neurobiological correlate across regular users of different substances (Noori et al. 2018). Interestingly, additional brain regions were implicated in both cannabis users and controls, during the cannabis cue-reactivity fMRI tasks (i.e. hippocampus, amygdala, thalamus, occipital cortex) in studies that did not include a control group. Functional activations of additional regions may be ascribed to salience processing, as images of illicit substances vs neutral stimuli, and may be more salient in both substance using and normative samples. Future work on picture rating of illicit substances vs neutral using controls is needed to confirm this notion. In sum, the neurobiological correlates of reactivity to cannabis-related cues in regular cannabis users may overlap with those implicated in (i) reward processing in cannabis users and (ii) reactivity to distinct substances in regular users of substances other than cannabis, a notion that is consistent with neuroscientific theories of addiction (Koob and Volkow 2016; Volkow and Morales 2015; Zehra et al. 2018).

Among correlations subjective craving was the most consistently examined and reported to be significant. Notably, the location of the region of which the activity correlated with craving, (partially) overlapped with that of areas with different activity between cannabis users and controls. Thus, altered function during cue-reactivity in these regions may drive higher self-reported subjective craving experienced as a result of cannabis cue exposure. These regions included the dorsal striatum, OFC and amygdala, and these regions are implicated in key aspects of cue-reactivity: habitual/compulsive use (Everitt 2014; Koob and Volkow 2016; Zehra et al. 2018), reward evaluation/motivational drive (Bechara 2005; Koob and Volkow 2016) and craving/stress levels, respectively (Koob and Volkow 2016; Zehra et al. 2018).

Emerging evidence from correlational analyses suggest that greater cannabis dependence and problems related with use, earlier cannabis use onset and comorbid tobacco use might be moderators of cue-reactivity-related functional brain alterations in regular cannabis users (Wetherill et al. 2016; Zhou et al. 2019). In a prospective study, cue-reactivity in the dorsal striatum was associated with cannabis dependence severity at 3-year follow-up, and cannabis dependence severity and subjective craving were also positively correlated (Vingerhoets et al. 2016). The findings from this review provides preliminary evidence, which is in-line with animal studies (Everitt 2014), and other substances of abuse (Jasinska et al. 2014), that the dorsal striatum may be a key brain region involved in cannabis dependence and cue-reactivity. Taken together, there is suggestion that the results may be driven by subgroups of cannabis users and explain some of the variance in the literature, which may include noise from inclusion of cannabis users with varying dependence severity (e.g. on the mild end of dependence) and cannabis use history (e.g. later age of onset). Future studies that include individuals with CUD on the more severe end and detailed reporting of cannabis use history are needed to examine this further.

Interestingly, the literature reported no association between brain function during cue-reactivity and measures of cannabis exposure (e.g. dosage, frequency). This is inconsistent with prominent neuroscientific theories of addiction which posit that neurobiological alterations in reward pathways occur with repeated substance exposure and related psychological correlates (e.g. tolerance, craving, withdrawal; Everitt 2014; Koob & Volkow 2016). This is also inconsistent with meta-analyses showing that greater cannabis dosage is associated with altered brain integrity (i.e. function and structure; Blest-Hopley et al. 2018; Rocchetti et al. 2013). It could be that exposure to cannabis is not consistently assessed across the reviewed studies and was examined by few studies, so this evidence might not be conclusive and needs to be corroborated by future work with sound assessment of cannabis exposure (e.g. detailed cannabis use history across the lifespan). Varying levels of cannabis exposure across the included samples prevent examination of this systematically as samples had cannabis users with different patterns of regular use (e.g. days/week) and varying level of exposure (grams/week). There may also be protective factors that preserve and/or moderate reward processing despite repeated cannabis exposure, such as age of onset, duration of use, treatment exposure and socio-economic status (Jasinska et al. 2014). Further, assessing cannabis potency/cannabinoid content is needed as different compounds (i.e. THC and CBD) have opposite effects on brain function (the latter being neuroprotective and former associated with psychotogenic effects), and these may conflate the results (Bhattacharyya et al. 2010).

Importantly, the design of the reviewed evidence was cross-sectional. Indeed, our review aimed to cross-sectionally compare brain function between cannabis users and controls, and the design of most studies to date was also cross-sectional. Thus, future longitudinal neuroimaging studies are warranted to investigate how the neurobiological correlates of cannabis cue-reactivity change over time. Specifically, a priority of future work is to determine if (i) functional alterations represent a neurobiological vulnerability that predates or predicts the onset of cannabis use and related problems, as greater sensitivity to reward has been implicated in increased risk of substance use and related problems (Everitt 2014); (ii) change over time with variations in the level of cannabis exposure and related problems (e.g. exacerbate with the progression to more chronic/severe CUD or mitigate with the transition to lighter forms of use); and (iii) dissipate or persist with abstinence from cannabis use.

The reviewed literature is limited by the use of inconsistent methodologies to measure cannabis use and cue-reactivity, and this issue prevents the direct integration of the study findings. First, the measurement of cannabis use and cannabis use-related problems occurred in limited studies and varied widely, and only a few studies ran correlations between brain function and cannabis use levels (e.g. distinct inclusion/exclusion criteria, use of different indices of exposure and over different periods of time, only two studies reported cannabinoids via toxicology analyses of biological specimens, four studies assessed duration of abstinence from cannabis and not in relation to brain function). Thus, an important area for future work is to use standardised measures of cannabis use. These include detailed measurement of current and lifetime use via timeline follow back methodologies (Sobell and Sobell 1992), which may clarify if frequency of use plays a role in cue-reactivity in cannabis users. Furthermore, detailed measurement of cannabis dosage, type, strength and method of uses via integrating to scale visual aids to the TLFB could investigate if these parameters of use drive reactivity to specific cannabis-related cues. For example, people who use cannabis via joints may experience greater reactivity when viewing images of joints, and this may direct clinicians to implement interventions to target reactivity to specific triggers of relapse. Additionally, a greater understanding of the role of craving and withdrawal on brain function in cannabis users would be achieved with reporting of abstinence duration at the time of data collection and with running correlations between abstinence duration and brain functional indices during cue-reactivity. Finally, to determine whether specific subgroups of cannabis users show more marked neural alterations during cue-reactivity, it would be useful to perform a clinical assessment of cannabis use-related problems that identifies the more vulnerable of users (e.g. presence and/or severity of CUD and of psychopathologies). Vulnerable cannabis users might include those with a more severe CUD or those using more potent and addictive cannabis varieties with high level of THC and low level of CBD with known distinct properties on brain function (Bhattacharyya et al. 2010) or people who have been abstinent from cannabis for longer time periods prior to scan. Such new knowledge is required to understand how brain functional alterations relate to clinical and public health issues.

Second, a comparison control group of non-cannabis users was used in less than half of the studies, and more evidence is required to confirm the location and direction of the group differences. Third, a meta-analysis could not be run as only one study (Zhou et al. 2019) met criteria for inclusion in a meta-analysis (i.e. reported all coordinates and utilised a whole brain approach; Müller et al. 2018). Future research is needed that employ methods and report details that allow for inclusion in meta-analysis to provide a systematic synthesis of findings to further our understanding of the neurobiology of reactivity to cannabis cues in regular cannabis users. Fourth, inconsistent methodologies were used to examine cue-reactivity, such as which stimuli were used as cues (e.g. modalities and matching of CAN and NEU), the fMRI cue-reactivity task design (e.g. duration, presentation order) and measurement of subjective craving (e.g. at different time points in relation to the fMRI task). Future research is required to use designs that allow for replicability of findings and their direct integration and to use and share via open access platforms, cannabis stimuli with stronger ecological validity (e.g. favourite product, people’s own cannabis and internal cues such as specific emotional states) which could be subsequently used to target in cue exposure therapy for the treatment of CUD.

Last, a major limitation of the literature is the lack of any analyses that explored associations between brain function during cue-reactivity and the severity of sub-clinical or diagnosed mental disorders that are commonly associated with cannabis use, dependence and greater reactivity to cannabis cues and cannabis craving (e.g. depression, anxiety, psychotic symptoms) (Meier et al. 2016) or of well-being measures associated with cannabis use (e.g. increased contact with peers, greater relaxation; Kilwein et al. 2020). Future work is warranted to embrace the systematic assessment of mental health and well-being in the cannabis using samples, so that the clinical significance of the literature findings can be appreciated.

### Clinical implications

In the context of CUDs, there is very limited neuroimaging research investigating cue-reactivity-targeted interventions. The findings from the literature can be used to inform the development of interventions designed to mitigate aberrant brain function associated with cue-reactivity in regular cannabis users. A reduction in brain cue-evoked activation in the amygdala and medial PFC (which are both implicated in cannabis cue-reactivity and subjective cannabis craving) has been reported after cognitive bias modification (CBM) relative to a sham-training control condition with alcohol-dependent participants, with the reduction significantly correlated with reduced subjective craving scores (Wiers et al. 2015). While there has only been one small pilot randomised controlled trial of cognitive bias modification (CBM) with cannabis users to date, those receiving the active intervention showed blunted cannabis cue-induced craving at the end of training compared to those in the sham-training controls, though greater reductions in cannabis use were only observed among male participants in the active condition (Sherman et al. 2018). Two pilot studies on mindfulness-based interventions showed a reduction in subjective craving and weekly cigarette dosage (mindfulness vs passive placebo) and in brain function during a cigarette cue-reactivity task pre-to-post intervention in cigarette smokers (i.e. ventral striatum, ACC, ventral and medial PFC; Westbrook et al. 2012). These regions are also associated with cannabis cue-reactivity in cannabis users as per this review. In sum, interventions such as CBM, ApBM and mindfulness-based interventions may be effective in reducing cue-reactivity and craving in users of substances other than cannabis, and future work is required to test this notion in regular cannabis users.

## Conclusions

Overall, the evidence points to greater brain function during cannabis cue-reactivity in regular cannabis users in specific brain pathways (striatal, PFC and parietal regions, followed by the hippocampus, amygdala and other regions), which might reflect a common neurobiology of altered reward processing across cannabis and other substances. Preliminary findings also show that greater brain function within such pathways (striatum, OFC and amygdala) may drive greater cannabis subjective craving in response to cannabis-related cue exposure and may not be relevant to cannabis use itself (i.e. no correlation between dosage and brain function). Our review also highlights the need for greater standardised assessment of the neurobiology of cue-reactivity in cannabis users, cannabis use and cannabis-use related problems and (sub-clinical and diagnosed) mental health problems. Finally, longitudinal studies are required to profile how brain function during cannabis cue-reactivity changes over time and in people as they develop greater severity of CUD, relapse or quit cannabis consumption. Overall, more robust fMRI evidence is required in order to fully determine the clinical relevance of altered brain function that cannabis users have in response to cannabis-related cues.

## Supplementary Information

Below is the link to the electronic supplementary material.

Supplementary file1 (DOC 358 KB)
