# Supplementary material for: Patterns of brain function associated with cannabis cue-reactivity in regular cannabis users: a systematic review of fMRI studies
Source: Psychopharmacology (Berl). 2021 Sep 10;238(10):2709–28. doi: 10.1007/s00213-021-05973-x (PMC8455486; doi:10.1007/s00213-021-05973-x)
Supplement: Supplementary file 1 — Supplementary file1 (DOC 358 KB) [file 213_2021_5973_MOESM1_ESM.doc]

**Patterns of brain function associated with cue-reactivity in regular cannabis users: A systematic review of fMRI studies**

Hannah Sehl^1^, Gill Terrett^1^, Lisa-Marie Greenwood^2, 3^, Magdalena Kowalczyk^1^, Hannah Thomson^1^, Govinda Poudel^4^, Victoria Manning^5^, Valentina Lorenzetti^1^

1. Neuroscience of Addiction & Mental Health Program, Healthy Brain and Mind Research Centre, School of Behavioural and Health Sciences, Faculty of Health Sciences, Australian Catholic University, Melbourne, Australia
2. Research School of Psychology, Australian National University, Canberra, Australia
3. The Australian Centre for Cannabinoid Clinical and Research Excellence (ACRE), New Lambton Heights, New South Wales, Australia
4. Mary Mackillop Institute for Health Research, Australian Catholic University, Melbourne
5. Turning Point, Eastern Health, Monash University, Melbourne, Australia

**Corresponding Author**

Valentina Lorenzetti, Neuroscience of Addiction & Mental Health Program, Healthy Brain and Mind Research Centre, School of Behavioural & Health Sciences, Faculty of Health Sciences, Australian Catholic University

A:  Daniel Mannix building, 17 Young Street, Fitzroy VIC 3065 Australia

T: (+61) 03 9230 8088

E: [valentina.lorenzetti@gmail.com](mailto:valentina.lorenzetti@gmail.com)

# Supplementary Material

## Overview of participants inclusion and exclusion criteria

### Overview of exclusion of psychopathologies

Ten of the eighteen studies excluded participants with a co-morbid DSM-4 axis-1 disorder other than cannabis abuse/dependence (Bitter et al. 2014; Charboneau et al. 2013; Cousijn et al. 2013; Goldman et al. 2013; Karoly et al. 2019; Vingerhoets et al. 2016; Wetherill et al. 2014; Wetherill et al. 2016; Wetherill et al. 2015; Zhou et al. 2019). Additional exclusion criteria included a lifetime history of psychotic symptoms (4 studies; de Sousa Fernandes Perna et al. 2017; Filbey and Dunlop 2014; Filbey et al. 2016; Filbey et al. 2009) or receiving a diagnosis of or treatment for schizophrenia or other psychotic disorder, bipolar disorder, or depression within the past 6 months (Kleinhans et al. 2020). Further, single studies excluded high levels of depression – i.e., Beck’s Depression Inventory scores > 20 (Beck et al. 1996), and known prenatal alcohol or illicit substance exposure (Karoly et al. 2019).

The reviewed studies used different criteria for excluding levels of comorbid illicit substance use, alcohol and nicotine. These included a positive uranalysis for any illicit substance (9 studies; Charboneau et al. 2013; Cousjin et al. 2013; Filbey et al. 2009; Filbey et al. 2014; Filbey et al. 2016; Karoly et al. 2019; Kuhns et al. 2020; Yoo et al. 2020, Zhou et al. 2019), self-report of a substance use disorder in the past 6-months plus any use of psychoactive substances in the past 4-weeks (2 studies; Goldman et al. 2013; Zhou et al. 2019), in the past 60 days (Yoo et al. 2020), current use of prescribed or illicit psychoactive drugs (Kuhns et al. 2020), > 100 lifetime occasions of any substance (other than cannabis; 2 studies; Karoly et al. 2019, Kuhns et al. 2020), and reported moderate -to-high risk use of other illicit substances (ASSIST Substance Involvement Score ≥ 4 for each substance reported; Kleinhans et al. 2020). Alcohol dependence was excluded using DSM-4 criteria (2 studies; Filbey et al. 2016, de Sousa Fernandes Perna et al. 2017), Alcohol Use Disorder Identification Test [AUDIT] scores > 10 (1 study; Cousjin et al. 2013) and scores > 12 (Kuhns et al. 2020), and CAGE score >2 (Kleinhans et al. 2020).

Eleven studies did not exclude any level of nicotine use (Bitter et al. 2014;; Charboneau et al. 2013; Feldstein Ewing et al. 2013; Filbey et al. 2009; Filbey et al. 2014; Goldman et al. 2013; Karoly et al. 2019; Wetherill et al. 2014; Wetherill et al. 2015; Wetherill et al. 2016, Yoo et al. 2020), whereas varying cut-off levels were reported across six studies – i.e., > 20 cigarettes/day (Cousjin et al. 2013; Kuhns et al. 2020, Vingerhoets et al. 2016; Zhou et al. 2019); > 15 cigarettes/day (de Sousa Fernandes Perna et al. 2017); > 1 pack/month (Filbey et al. 2016;) One study reported screening IQ > 79 (Goldman et al. 2013). A single study screened for history of learning or developmental disability (Karoly et al. 2019).

### Overview of Exclusions of Medical-Related Criteria

Majority of the included studies (except 5 studies; Feldstein Ewing et al. 2013; Filbey et al. 2009; Filbey et al. 2014; Filbey et al. 2016, Kleinhans et al. 2020) excluded participants with an unstable/major medical condition or neurological disorder. Similarly, history of traumatic brain injury was excluded in all but seven studies (Cousjin et al. 2013; Filbey et al. 2009; Kleinhans et al. 2020, Kuhns et al. 2020, de Sousa Fernandes Perna et al. 2017; Vingerhoets et al. 2016; Zhou et al. 2019). Use of medications were excluded if they affect the central nervous system (5 studies; Kleinhans et al. 2020, Wetherill et al. 2014; Wetherill et al. 2015; Wetherill et al. 2016; Zhou et al. 2019), classed as psychotropic (4 studies; Bitter et al. 2014; Charboneau et al. 2013; Kleinhans et al. 2020, de Sousa Fernandes Perna et al. 2017), anti-psychotic or anti-convulsant (1 study; Feldstein Ewing et al. 2013), and vasoactive (1 study; Charboneau et al. 2013). Other medical exclusions reported by single studies were cardiovascular abnormalities, hypertension, unhealthy BMI (de Sousa Fernandes Perna et al. 2017), hyposmia, anosmia (Kleinhans et al. 2020), regular exercise > 2-hours/week over the past month (Charboneau et al. 2013), a new tattoo in the past month (Feldstein Ewing et al. 2013), and premature or low birth weight and sensory problems (Karoly et al. 2019). All studies screened for MRI contraindications (e.g. floating metal in body), and studies comprising of female participants screened out individuals who were pregnant or breastfeeding.

### Overview of Inclusion Criteria for Types of Groups

Criteria related to cannabis use levels for inclusion in the cannabis, cannabis subgroups, and non-using control groups varied across the reviewed studies and have been summarised below.

#### *Cannabis* *Group*

In eight of the 18 studies, inclusion in the cannabis sample was based on meeting DSM-4 criteria for cannabis abuse (2 studies; Charboneau et al. 2013; Filbey et al. 2014), or cannabis dependence (5 studies; Bitter et al. 2014; Goldman et al. 2013; Wetherill et al. 2014; Wetherill et al. 2015; Wetherill et al. 2016), with one study specifying a minimum length of time (i.e., past 12-months; Bitter et al. 2014). Eleven of the eighteen studies set specific cannabis use patterns as inclusion criteria which varied across studies (Cousjin et al. 2013; Feldstein Ewing et al. 2013; Filbey et al. 2009; Filbey et al. 2014; Filbey et al. 2016; Goldman et al. 2013; Karoly et al. 2019; Kuhns et al. 2020; de Sousa Fernandes Perna et al. 2017; Yoo et al. 2020, Wetherill et al. 2014), four of which were in addition to DSM-4 abuse/dependence criteria being met (Bitter et al. 2014; Filbey et al. 2014; Goldman et al. 2013; Wetherill et al. 2015). A single study screened out cannabis users that had a current and/or history of cannabis abuse/dependence (Cousjin et al. 2013), and another did not report on initial inclusion criteria for their cannabis group (Zhou et al. 2019). One study used Cannabis subtest of the Alcohol, Smoking and Substance Involvement Screening Test (ASSIST) to determine the inclusion in the cannabis dependence group (ASSIST Substance Involvement Score ≥ 4; Kleinhans et al. 2020),

Eleven studies included non-treatment seeking cannabis users (Bitter et al. 2014; Charboneau et al. 2013; Cousjin et al. 2013; Feldstein Ewing et al. 2013; Filbey et al. 2009; Filbey et al. 2014; Filbey et al. 2016; Goldman et al. 2013; Kuhns et al. 2020 Yoo et al. 2020, Wetherill et al. 2014), five studies recruited treatment seeking samples (Karoly et al. 2019; Wetherill et al. 2014; Wetherill et al. 2015; Wetherill et al. 2016; Zhou et al. 2019), and two studies did not report on treatment status (de Sousa Fernandes Perna et al. 2017, Kleinhans et al. 2020). The minimum period of abstinence from cannabis for inclusion varied across studies from 0-hours/normal use (1 study; Goldman et al. 2013), 12-hours (1 study; Karoly et al. 2019), 24-hours (8 studies; Bitter et al. 2014; Cousjin et al. 2013; Feldstein Ewing et al. 2013; Kuhns et al. 2020 Wetherill et al. 2014; Wetherill et al. 2015; Wetherill et al. 2016; Zhou et al. 2019), 48-hours (study, Kleinhans et al. 2020), 72-hours (4 studies; Filbey et al. 2009; Filbey et al. 2014; Filbey et al. 2016, Yoo, et al. 2020) to 168-hours (1 study; de Sousa Fernandes Perna et al. 2017).

#### *Cannabis* *Subgroups*

***Dependent vs Non-Dependent Cannabis Users.*** Two of the 18 studies performed a sub-group analysis comparing dependent and non-dependent cannabis users (Filbey et al. 2014; Zhou et al. 2019). One analysed brain activity (Zhou et al. 2019), and the other, brain connectivity (Filbey et al. 2014). Both studies characterised dependence according to DSM-4 criteria using the Structured Clinical Interview for DSM-4- (SCID-4; Filbey et al. 2014) and Mini International Neuropsychiatric Interview (MINI; Zhou et al. 2019).

***High-Problem vs Low-Problem Cannabis Users.*** A single study carried out a sub-group analysis comparing cannabis users characterised as high-problem users versus low-problem users according to their Cannabis Use Identification Test (CUDIT) scores (Cousjin et al. 2013).

***Frequent vs Sporadic Cannabis Users.*** Two groups were included in a study based on their cannabis use patterns. Frequent was defined as cannabis use > 10-days/month for at least 2-years, whereas sporadic cannabis use was defined as 1-50 lifetime occasions (Cousjin et al. 2013).

***Early Onset vs Late Onset Cannabis Users.*** One study compared dependent cannabis users based on their age of onset. Participants were grouped as early onset if their cannabis use commenced < 16 years old and late onset if their use started > 16 years old (Wetherill et al. 2016).

***Male vs Female Cannabis Users.*** In one study cannabis users were compared based on their sex (male and female; Wetherill et al. 2015). Of the 44 participants, 20 were also included in the study conducted by Wetherall et al., 2014, which performed a within group analysis of dependent cannabis users.

***Baseline vs 3-year Follow-up Frequent Cannabis Users.*** A single study examined associations between frequent cannabis baseline measures of brain function during a fMRI cue-reactivity task with cannabis use patterns at 3-year follow-up (Vingerhoets et al. 2016).

***Co-users of cannabis and cigarettes vs users of cannabis only.***  A single study compared cannabis users and co-users of cannabis and tobacco (Kuhns et al. 2020)

#### *Non-Using Control Group*

Seven of the 18 studies included a non-using control group (Bitter et al. 2014; Cousjin et al. 2013; Filbey et al. 2016; Kleinhans et al. 2020, de Sousa Fernandes Perna et al. 2017; Yoo et al. 2020, Zhou et al. 2019). The non-using control groups were included and excluded against the same criteria as the cannabis groups, with the exception of their cannabis use which varied across all six studies. One study excluded participants if their lifetime cannabis use exceeded 10 occasions and/or 15 grams (Zhou et al. 2019). Another study screened for any lifetime occasions of cannabis use (Cousjin et al. 2013), whereas one excluded individuals with any lifetime period of daily use (Filbey et al. 2016). One study included controls based on the absence of daily cannabis use at any period in their lifetime, as well as an absence of current illicit drug use in the past 60 days (Yoo et al. 2020). A single study compared users of cannabis only and co-users of cannabis and tobacco with controls using only tobacco or not (Kuhns et al. 2020). In this study controls were allowed to have used cannabis up to 50 times in their life, but not during the past year (Kuhns et al. 2020). One study did not report these details (Bitter et al. 2014;).

### Overview of Substance Use Assessment

#### *Assessment of Substance Use Levels*

Majority of the studies measured the quantity (e.g. grams, cigarettes, drinks), frequency (e.g. number of days, occasions), and duration (e.g. years, age of onset) of cannabis (18 studies), alcohol (16 studies), nicotine (13 studies), and other illicit substance use (2 studies; Charboneau et al. 2013; Zhou et al. 2019) using the Timeline Follow Back (TLFB) calendar (Sobell and Sobell 1992) or a similar self-report method.

Presence and/or levels of recent substance use was assessed via urine sample (10 studies; Charboneau et al. 2013; Cousjin et al. 2013; Filbey et al. 2009; Filbey et al. 2014; Filbey et al. 2016; Karoly et al. 2019; Kuhns et al. 2020 Zhou et al. 2019, Yoo et al. 2020), breath (2 studies; Charboneau et al. 2013; Karoly et al. 2019), and blood (1 study; de Sousa Fernandes Perna et al. 2017).

#### *Assessment of Substance Dependence Severity*

All studies (except two; de Sousa Fernandes Perna et al. 2017; Karoly et al. 2019) assessed cannabis dependence severity levels. Cannabis dependence severity was verified via a variety of semi-structured clinical interviews and questionnaires. Semi-structured clinical interviews included the MINI (4 studies, Charboneau et al. 2013; Goldman et al. 2013; Vingerhoets et al. 2016; Zhou et al. 2019), the SCID-4 (3 studies; Filbey et al. 2009; Filbey et al. 2014; Filbey et al. 2016); and the Addiction Severity Index (ASI; 4 studies; Bitter et al. 2014; Wetherill et al. 2014; Wetherill et al. 2015; Wetherill et al. 2016). Self-report measures included the CUDIT (4 studies; Cousjin et al. 2013; Kleinhans et al. 2020, Kuhns et al. 2020, Vingerhoets et al. 2016), Marijuana Dependence Scale, and Cannabis Problems Questionnaire-Adolescents (1 study; Feldstein Ewing et al. 2013).

Alcohol dependence severity was assessed in nine of 18 studies (Bitter et al. 2014; Charboneau et al. 2013; Cousjin et al. 2013; Filbey et al. 2016; Kleinhans et al. 2020, Vingerhoets et al. 2016; Wetherill et al. 2014; Wetherill et al. 2015; Wetherill et al. 2016). Four used the Semi-Structured Assessment for the Genetics of Alcoholism, Adolescent Version (Bitter et al. 2014; Wetherill et al. 2014; Wetherill et al. 2015; Wetherill et al. 2016), four used the Alcohol Use Identification Test (AUDIT; Cousjin et al. 2013; Kleinhans et al. 2020, Kuhns et al. 2020, Vingerhoets et al. 2016), and single studies used the SCID (Filbey et al. 2016) and the MINI (Charboneau et al. 2013).

Nicotine dependence severity was assessed by six of 18 studies. Four used the Fagerstrom Test for Nicotine Dependence (FTND; Cousjin et al. 2013; Kuhns et al. 2020, Vingerhoets et al. 2016; Wetherill et al. 2014), single studies used the SCID (Filbey et al. 2016), the ASI (Wetherill et al. 2016), and the ASSIST—Tobacco (Kleinhans et al. 2020).

### Overview of Group Matching (Cannabis vs Control and Sub-Groups).

In studies that had a non-using control (8 studies; Bitter et al. 2014; Cousjin et al. 2013; de Sousa Fernandes Perna et al. 2017; Filbey et al. 2016; Kleinhans et al. 2020, Kuhns et al. 2020, ; Zhou et al. 2019) groups were matched on age and sex, and one study on age only (de Sousa Fernandes Perna et al. 2017). Groups were matched on IQ in three studies (Bitter et al. 2014; Cousjin et al. 2013, Kuhns et al. 2020), and cannabis users had lower IQ than controls in one study (Bitter et al. 2014). Similarly, in single studies groups were matched in years of education (Zhou et a., 2019), with one cannabis group having less years of education than non-using controls (Filbey et al. 2016). Where reported, dependent versus non-dependent cannabis using samples in two studies were matched on age, sex, IQ, years of education, duration of cannabis use (years), and lifetime cannabis (grams). However, in one study the dependent group had an earlier age of onset than the non-dependent (Zhou et al. 2019), and in the other, the non-dependent group smoked more cigarettes/day than the dependent group. High-problem and low-problem groups were matched across reported demographics except for levels of problems (Cousjin et al. 2013). Frequent and sporadic cannabis users had matching demographics, however frequent users reported smoking more cigarettes/day, longer lifetime cigarette smoking and greater nicotine dependence (Cousjin et al. 2013). In one study that compared male and female samples (Wetherill et al. 2015), groups were matched on reported demographics other than sex. One study reported matching on alcohol use and problems, other substance use, anxiety, depression, ADHD (Kuhns et al. 2020).

### Overview of subjective craving ratings during the cue-reactivity fMRI task

Supplementary Table 1 overviews the measures used to rate participants’ subjective level of cannabis craving during the cue-reactivity fMRI task, when these measures were administered, and any changes in subjective craving in relation to cannabis and neutral cues. The relevant results are summarised below.

### Overview of self report measures used to rate craving

Two measures were used to rate craving during the cue-reactivity fMRI task. Most studies measured self-reported subjective craving using either Marijuana Craving Questionnaire (MCQ; 6 studies Filbey et al. 2009, Filbey et al. 2014; Karoly et al. 2019; Kleinhans et al. 2020; Wetherill et al. 2015; Zhou et al. 2019), or the Visual Analogue Scale (VAS; 6 studies; Bitter et al. 2014; Charboneau et al. 2013; Cousijn et al. 2013; Filbey et al. 2016; Kuhns et al. 2020; Vingerhoets et al. 2016). Two studies used both the MCQ and VAS (Goldman et al. 2013; Yoo et al. 2020;), and four studies did not report the measure (de Sousa Fernandes Perna et al. 2017; Feldstein Ewing et al. 2013; Wetherill et al. 2015; Wetherill et al. 2016). Craving was measured either both pre and post the fMRI task (8 studies), or after every stimulus in the fMRI task (6 studies).

### Overview of self-reported level of craving in the samples

Changes in cannabis users’ self report craving assessed pre and post the fMRI task, showed increased in craving in seven studies and not change in craving in three studies. Other four studies reported that participants’ that craving levels in relation to cannabis stimuli were higher than those reported in relation to neutral stimuli.

## Overview of fMRI cue-reactivity task stimuli

Supplementary Table 2 overviews the characteristics of the stimuli used in the literature as cues during the cue-reactivity fMRI tasks, which were described in all studies but one (Bitter et al. 2014). *Cannabis related cues* included different types of stimuli: images in 11 studies (e.g., showing paraphernalia [e.g. pipe, joint], cannabis plant matter, and people using/holding cannabis); images *and* tactile stimuli (e.g., pipe) to be held by participants in the scanner in 5 studies,; images and odor in one study, and audio-visual marketing clips in one study. *Neutral cues* (i.e. defined as non-cannabis, non-rewarding cues) included different types of stimuli: images (e.g., of people interacting with objects, nature, building facades, stationary, cars and faces). Cannabis and control stimuli were *matched* for basic perceptual features in all but two studies (de Sousa Fernandes Perna et al. 2017; Zhou et al. 2019), and three more studies did not provide any information on matching (Bitter et al. 2014; Kleinhans et al, 2020, Kuhns et al. 2020). Eight studies included *additional control stimuli*: rewarding images (related to food and sex), aversive images, control images (blurred, animals), and alcohol marketing clips (Charboneau et al. 2013; Cousijn et al. 2013; Filbey et al. 2016; Karoly et al. 2019; de Sousa Fernandes Perna et al. 2017; Vingerhoets et al. 2016; Wetherill et al. 2014; Wetherill et al. 2015).

## Overview of the methodological characteristics of the cue-reactivity fMRI task

Supplementary Table 3 overviews the design and characteristics of the cue-reactivity fMRI tasks (e.g., duration, number of stimuli, runs, blocks, stimuli/block, presence of a fixation cross). Most studies used a *block design* (n = 11), and the others used an *event-related* design (n = 7). The *duration* of the fMRI cue-reactivity task ranged from 8 to 33 minutes (average of 15 minutes) and varied widely with no more than four studies using a task of the same duration. The *order of stimulus presentation* was pseudo, -random or quasi order the studies. All but three studies presented cannabis stimuli first (Bitter et al. 2014; Filbey et al. 2009; Yoo et al. 2020). Other fMRI task characteristics varied widely between studies: i) the *number of stimuli* (cannabis and neutral) ranged from 1-to-96, ii) the *number of task runs* varied between 1 and 3; iii) there were 2-to-24 *blocks*; iv) the *number of stimuli used per block* ranged from 5 to 96, and v) the duration of stimulus’ presentation was 0.33 to 20 seconds. Most studies presented a *fixation cross* between stimuli for a heterogeneous duration (from 0.5-20 seconds), and a rest between blocks for 20 seconds.

**Supplementary Table 1**

*Overview of measures and outcomes of self-reported Craving during the Cue-Reactivity fMRI Task*

| Author, year | Self-Reported Craving Rating | | | |
| --- | --- | --- | --- | --- |
|  | Measure | Time Administered | Pre vs Post cue reactivity fMRI Task | |
|  |  |  | Cannabis | Control |
| Filbey, 2009 | VAS (0-10) | Post each cue | CAN > NEU (craving) | _ |
| Charboneau, 2013 | MCQ | Pre & Post MRI | Post > Pre | _ |
| Cousijn, 2013 | MCQ | Pre & Post MRI | Post > Pre | _ |
| Feldstein Ewing, 2013 | _ | _ | _ | _ |
| Goldman, 2013 | VAS (0-10) | Post each cue | Post = Pre | _ |
| Bitter, 2014 | MCQ | Pre & Post MRI | Post = Pre | Post = Pre |
| Filbey, 2014 | VAS (0-10) | Post each cue | CAN > NEU (craving) | _ |
| Wetherill, 2014 | _ | _ | _ | _ |
| Filbey, 2016 | MCQ | Pre & Post MRI | Post = Pre | Post = Pre |
| Wetherill, 2015 | VAS (0-10) | Post each cue | CAN > NEU (craving) | _ |
| de Sousa Fernandes Perna, 2017 | _ | _ | _ | _ |
| Vingerhoets, 2016 | MCQ | Pre & Post MRI | Post > Pre | _ |
| Wetherill, 2016 | _ | _ | _ | _ |
| Karoly, 2019 | VAS (0-5) | Post each block | CAN > NEU (wanting) | _ |
| Zhou, 2019 | VAS (0-100) | Pre & Post MRI | Post > Pre | _ |
| Kuhns, 2020 | MCQ | Pre & Post MRI | Post > Pre | Post = Pre |
| Yoo, 2020 | VAS (0-10), MCQ | Post each cue | Post > Pre | _ |
| Kleinhans, 2020 | VAS (0-10) | Pre & Post MRI | Post > Pre | Post = Pre |

Note: VAS = Visual Analogue Scale, MCQ = Marijuana Craving Questionnaire, CAN = Cannabis Stimuli, NEU = Neutral Stimuli; ^*^ Not analysed for significance

**Supplementary Table 2.**

*Overview of type of cannabis and neutral stimuli presented as cues during the fMRI Cue-Reactivity Task*

|  | Type | Cannabis | | Neutral | | Control | |
| --- | --- | --- | --- | --- | --- | --- | --- |
|  |  | Source | Content Example | Source | Content Example | Source | Content Example |
| Filbey,  2009 | Tactile pipe & mirrored image | _ | Pipe | _ | Pencil | _ | _ |
| Charboneau, 2013 | Image | _ | Close-up whole plant, dried, joints, bong, pipe, papers with/without people | _ | Landscapes, animals, or insects both close-up & far away | _ | Food;  Gaussian blurred |
| Cousijn,  2013 | Image | a | Whole plant, dried, joints, bong, pipe, papers with/without people | a | Individuals & objects | a | Animals |
| Feldstein Ewing, 2013 | Tactile pipe & mirrored image | _ | Pipe | _ | Pencil | _ | _ |
| Goldman,  2013 | Image | _ | Whole plant, dried, joints, bong, pipe, papers with/without people | _ | Stationary, keys | _ | _ |
| Bitter,  2014 | Image | Public | Whole plant, dried | IAPS | Faces, cars, nature | _ | _ |
| Filbey,  2014 | Tactile pipe & mirrored image | _ | Pipe | _ | Pencil | _ | _ |
| Wetherill,  2014 | Image | _ | Whole plant, dried, joints, bong, pipe, papers with/without people | Authors laboratory archive | Building facades, people engaged in everyday activities | IAPS | Sexual & aversive images |
| Wetherill,  2015 | Image | _ | Whole plant, dried, joints, bong, pipe, papers with/without people | Authors laboratory archive | Building facades, people engaged in everyday activities | IAPS | Sexual & aversive images |
| Filbey,  2016 | Tactile method & mirrored image | _ | Preferred method of use (e.g. pipe, bong, blunt, joint) | _ | Pencil | Fruit  (preferred) | Tactile piece of fruit & mirrored image |
| Vingerhoets,  2016 | Image | b | Whole plant, dried, joints, bong, pipe, papers with/without people | b | Individuals & objects | b | Animals |
| Wetherill,  2016 | Image | _ | Whole plant, dried, joints, bong, pipe, papers with/without people | Authors laboratory archive | Building facades, people engaged in everyday activities | _ | _ |
| de Sousa Fernandes Perna, 2017 | Audio-visual clip | _ | CB marketing clips included adverts for CB paraphernalia & a selection of short film fragments portraying CB use & marketing practices at CB selling points | _ | Not described |  | Alcohol marketing clips |
| Karoly,  2019 | Image | _ | Whole plant, dried, joints, bong, pipe, papers with/without people | _ | Non-food objects and plants | _ | Blurred images |
| Zhou,  2019 | Image | c | Whole plant, dried, joints, bong, pipe, papers with/without people | IAPS + NAPS | *Not described* |  |  |
| Kuhns, 2020 | Image | c | flower nuggets, joints, and individuals smoking cannabis. | c | Office supplies | *Not described* | *Not described* |
| Yao,  2020 | Tactile method & mirrored image | Preferred method of use (e.g. pipe, bong, blunt, joint) | _ | Pencil | _ | Fruit  (preferred) | Tactile piece of fruit & mirrored image |
| Kleinhans, 2020 | Image + odor | _ | paraphernalia, the cannabis odorant, non-psychoactive garden-variety flowers and related products, pure phenylethyl alcohol which smells like roses | _ | Cross | *Not described* | *Not described* |

Note: IAPS = International Affective Picture System (Lang et al. 1988), NAPS = The Nencki Affective Picture System (Marchewka et al. 2014).

^a^ Adapted (McClernon et al. 2005)

^b^ Same task as Cousijn et al. 2013

^c^ Adapted from Cousijn et al. 2013

**Supplementary Table 3**

*fMRI Cue-Reactivity Task Duration, Design, and Stimuli Presentation Protocol*

| 1^st^ author, year | Total task duration | Task Design | Stimuli, *n* | | Runs, *n* | Blocks, *n* | Stimuli shown/Block, *n* | Stimulus Presentation, *sec* | Inter Stimulus Interval | | Rest between Blocks, *sec* | Random Stimuli Presentation | VAS presentation, *sec* |
| --- | --- | --- | --- | --- | --- | --- | --- | --- | --- | --- | --- | --- | --- |
|  |  |  | Cannabis | Neutral |  |  |  |  | Duration, *sec* | Fixation cross |  |  |  |
| Filbey,  2009 | 19 min & 12 sec | Block | 1 | 1 | 2 | 2 | 6 | 20 | 20 | ✓ | 20 | Pseudo | 5 |
| Charboneau, 2013 | ~9 min | Block | 30 | 30 | 3 | 6 | 10 | 3 | _ | _ | _ | Random | _ |
| Cousijn,  2013 | 11 min | Event-related | 30 | 30 | 1 | 1 | 75 | 4 | jittered b/n 2-6 | ✓ | _ | Quasi | _ |
| Feldstein Ewing, 2013 | ~21 min & 20 sec | Block | 1 | 1 | 1 | 4 | 5 | 20 | 20 | ✓ | 20 | Pseudo | 5 |
| Goldman,  2013 | 8 min | Block | 60 | 60 | 1 | 12 | 10 | 1.5 | 0.5 | ✓ | 20 | Semi ^a^ | _ |
| Bitter, 2014 | _ | Block | 6 | 6 | 1 | 5 | 6 | 4.75 | 2.5 | ✓ | 20 | Quasi | _ |
| Filbey,  2014 | 19 min & 12 sec | Block | 1 | 1 | 2 | 2 | 6 | 20 | 20 | ✓ | 20 | Pseudo | 5 |
| Wetherill,  2014 | 8.5 min | Event-related | 96 | 33 | 2 | 1 | 96 | 0.33 | 10-to-20 *jittered* | ✓ | _ | Random / quasi | _ |
| Wetherill,  2015 | 8.5 min | Event-related | 96 | 33 | 2 | 1 | 96 | 0.33 | 10-to-20 *jittered* | ✓ | _ | Random / quasi | _ |
| Filbey,  2016 | 28 min | Block | 1 | 1 | 3 | 3 | 6 | 20 | 20 | ✓ | 20 | Pseudo | 5 |
| de Sousa Fernandes Perna, 2017 | 33 min | Block | 10 | 10 | 1 | 30 sec blocks | _ | _ | _ | _ | _ | Random | _ |
| Vingerhoets, 2016 | 11 min | Event-related | 30 | 30 | 1 | 1 | 75 | 4 | 2-to-6 *jittered* | ✓ | _ | Quasi | _ |
| Wetherill,  2016 | 8.5 min | Event-related | 96 | 33 | 2 | 1 | 96 | 0.33 | 10-to-20 *jittered* | ✓ | _ | Random / quasi | _ |
| Karoly,  2019 | _ | Block | 36 | 36 | 1 | 24 | 6 | 6 | _ | _ | _ | Pseudo | 6 |
| Zhou,  2019 | _ | Block | 45 | 45 | 1 | 9 | 5 | 3 | 0.5-1.5 & 14.5-15.5 inter-block | ✓ | _ | Random | 5 |
| Kuhns, 2020 | _ | Event-related | 10 | 10 | 2 | 1 | _ | 4 | 2-to-6 *jittered* | ✓ | _ | Quasi | _ |
| Yao,  2020 ^b^ | 13 min & 30 sec (x 2) | Block | 1 | 1 | 2 | 12 | _ | 20 | _ | _ | _ | Pseudo | 5 |
| Kleinhans, 2020 | 12 min & 10 sec | Event-related | 77 | 77 | 1 | _ | _ | 0.85 | 0.25 | ✓ | _ | _ | _ |

Note: min = minute, sec = second, VAS = Visual Analogue Scale, N = number, Ctrl = Control Stimuli.

^a^ as per Gellermann series (Gellermann, 1933).

^b^ cue exposure task originally described in Filbey et al. (2016), which was modified from Filbey et al. (2009)

**Supplementary Table 4.**

*Technical characteristics of imaging data acquisition*

| **Author, Year** | **MRI scanner** | **N head coil’ channels** | **T1 acquisition parameters** | **fMRI acquisition parameters** |
| --- | --- | --- | --- | --- |
| Filbey,  2009 | 3T Siemens Trio | *NA* | MPRAGE, TR= 2300 ms, TE = 2.74 ms, TI = 900 ms, slab thickness = 176 mm, FOV =256 x 256 mm, matrix = 256 x 256 x 176, voxel size = 1 x 1 x 1 mm, number of echos = 4, pixel bandwidth = 650 Hz | EPI, TR = 2000 ms, TE = 27 ms, 32 slices, matrix size = 64 x 64, voxel size = 3 x 3 x 4 mm, FA = 70° |
| Charboneau,  2013 | 3T Philips Intera Achieva | *NA* | *NA* | *NA* |
| Cousijn,  2013 | 3T Philips Intera Achieva | 8 | TFE, TR= 9600 ms, TE = 4.6 ms, 182 slices, slice thickness =1.2 mm, FOV = 256 x 256 mm, in-plane resolution = 256 x 256, FA = 8° | EPI, TR = 2290 ms, TE = 30 ms, 38 slices, slice thickness = 3 mm, interslice gap = 0.3 mm, FOV = 220 x 220 mm, in-plane resolution = 96 x 96, FA = 80° |
| Feldstein Ewing, 2013 | 3T Siemens Trio | 12 | MPRAGE, TR = 2300 ms, TE = 2.74ms, TI = 900 ms, FOV = 256 x 256 mm, slab thickness = 176 mm, matrix = 256 x 256 x 176, voxel size = 1 x 1 x 1 mm, FA = 8°, number of echos = 4, pixel bandwidth = 650 Hz | EPI, TR = 2000 ms, TE = 27 ms, 32 slices, matrix size = 64 x 64, voxel size = 3 x 3 x 4 mm, FA = 70° |
| Goldman,  2013 | 3T Siemens Trio | *NA* | MPRAGE, TR = 1620ms, TE = 3 ms, FOV = 250 x 250 mm, matrix = 192 x 256, slice thickness = 1mm | EPI, TR = 2000 ms, TE = 30 ms, 33 slices, slices thickness = 3mm (no gap), FOV = 192 mm, matrix = 64 x 64  FA = 90° |
| Bitter,  2014 | 4T Varian Unity Inova | *NA* | *NA* | EPI, TR = 2000 ms |
| Filbey,  2014 | 3T Siemens Trio | 12 | MPRAGE, TR = 2300 ms, TE = 2.74 ms, TI = 900 ms, 192 slices, FOV = 256 x 256 mm, slab thickness = 176 mm, matrix = 256 x 256 x 176, voxel size = 1 x 1 x 1 mm, FA = 8°, number of echos = 4, pixel bandwidth = 650 Hz | EPI, TR = 2000 ms, TE = 27 ms, 32 slices, matrix = 64 x 64, voxel size = 3 x 3 x 4 mm,  FA = 70° |
| Wetherill,  2014 | 3T Siemens Trio | 8 | MPRAGE, TR = 510 ms, TE = 3.7 ms, 160 slices, FOV = 192 × 256 mm, slice thickness = 1 mm, FA = 90° | EPI, TR = 2000 ms, TE =  30 ms, 32 slices, slice thickness = 4.5 mm, FOV = 64 × 64 mm, FA =  90° |
| Wetherill,  2015 | 3T Siemens Trio | 8 | MPRAGE, TR = 510 ms, TE = 3.7 ms, 160 slices, FOV = 192 × 256 mm, slice thickness = 1 mm, FA = 90° | EPI, TR = 2,000 ms, TE = 30 ms, 32 slices, slice  thickness = 4.5 mm. FOV = 64 x 64 mm, FA = 90° |
| Filbey,  2016 | 3T Philips | *NA* | MPRAGE, TR = 8.2 ms, TE = 3.7 ms, TI = 1100ms,  FOV = 256 x 256 mm, slab thickness = 160 mm, voxel size = 1 x 1 x 1 mm, FA = 12° | EPI, TR = 2000 ms, TE = 29 ms, 39 slices, matrix = 64 x 64, voxel size = 3.44 x 3.44 x 3.5 mm, FA = 75° |
| de Sousa Fernandes Perna, 2017 | 3 T Siemens Magnetom Allegra | *NA* | MPRAGE, TR = 9.7 ms, TE = 4 ms, matrix = 256 ×256,  voxel size = 1 × 1 × 1 mm, FA = 12° | EPI, TR =2000 ms, TE= 30 ms, FOV = 224 mm, matrix = 64 x 64, voxel size = 3.5 x 3.5 x 3.5 mm, FA = 90° |
| Wetherill,  2016 | *NA* | *NA* | *NA* | *NA* |
| Karoly,  2019 | 3T Siemens Trio | *NA* | *NA* | EPI, TR = 2200 ms, TE = 35 ms, 37 slices, FOV = 192 mm, slice thickness = 3 mm,  Matrix = 64 × 64, voxel size = 3 × 3 mm, FA =90°; |
| Zhou,  2019 | 3T Siemens Trio | *NA* | TR = 1660 ms, TE = 2.54 ms, 208 slices, FOV = 256 mm, voxel size = 0.8 × 0.8 × 0.8 mm | EPI, TR = 2500 ms, TE = 30 ms, 37 slices, FOV = 192 mm, voxel size = 2 × 2 × 3 mm, FA = 90° |
| Kuhns, 2020 | 3T Philips Intera Achieva | 32 | TFE, TR = 8200 ms, TE = 3.8 ms, 220 slices, FOV = 240 × 188 mm, slice thickness = 1 mm, voxel size = 1 × 1 × 1 mm,  FA = 8° | EPI, TR = 2000 ms, TE = 27.63 ms, 37 slices, FOV =  240 × 240 mm, slice thickness =  3 mm, voxel size = 3 × 3 × 3 mm, slice gap = 3 mm,  flip angle = 76.1° |
| Yoo, 2020 | 3T Philips | *NA* | MPRAGE, TR = 8.1 ms, TE = 3.7 ms, voxel size = 1 × 1 × 1 mm, matrix = 256 × 256, FOV = 256 × 256 mm, FA = 12° | EPI, TR = 2000 ms, TE = 29 ms, FOV = 220 × 220 mm, matrix = 64 x 64, voxel size = 3.44 × 3.44 × 3.50 mm, FA = 75° |
| Kleinhans, 2020 | 3T Philips Intera Achieva | 32 | MPRAGE, TR = 7.6 ms, TE = 3.6 ms, TI = 910.5 ms,  FOV = 256 × 256 × 176 mm, matrix = 176 × 256  matrix, voxel size = 1 × 1 × 1 mm, FA = 7° | EPI, TR = 2000 ms, TE = 24 ms, 39 slices (no gap), FOV = 240 × 240 × 156 mm, matrix = 80 × 78, voxel  size = 3 × 3 × 4 mm, FA = 79° |

Note: EPI = echo-planar imaging, TR = repetition time, TE = echo time, TI = inversion time, FOV = field of view, FA = flip angle, TFE = Turbo Field Echo, MPRAGE = magnetization-prepared gradient echo, *NA* = not available

**Supplementary Table 5**

*Overview of the Risk of Bias: National Heart, Lung, and Blood Institute – Quality Assessment Tool for Observational Cohort and Cross-Sectional Studies*

| **Author, year** | **1** | **2** | **3** | **4a** | **4b** | **5** | **6** | **7** | **8** | **9** | **10** | **11** | **12** | **13** | **14** |
| --- | --- | --- | --- | --- | --- | --- | --- | --- | --- | --- | --- | --- | --- | --- | --- |
| Filbey, 2009 | Y | Y | Y | Y | Y | ***N*** | Y | Y | n/a | Y | n/a | Y | ***N*** | n/a | Y (cannabis dependence severity, cannabis problems, cannabis dosage, frequency, duration, age of onset) |
| Charboneau, 2013 | Y | Y | Y | Y | Y | ***N*** | Y | Y | n/a | Y | n/a | Y | ***N*** | n/a | ***N*** |
| Cousijn, 2013 | Y | Y | Y | Y | Y | ***N*** | Y | Y | n/a | Y | n/a | Y | ***N*** | n/a | Y (cannabis problems, cannabis lifetime use, cigarette/day) |
| Feldstein Ewing, 2013 | Y | Y | Y | Y | Y | ***N*** | Y | Y | n/a | Y | n/a | Y | ***N*** | n/a | Y (Age, gender, IQ, alcohol frequency) |
| Goldman, 2013 | Y | Y | Y | Y | Y | ***N*** | Y | Y | n/a | Y | n/a | Y | ***N*** | n/a | ***N*** |
| Bitter, 2014 | Y | Y | Y | Y | Y | ***N*** | Y | Y | n/a | Y | n/a | Y | ***N*** | n/a | Y (IQ) |
| Filbey, 2014 | Y | Y | Y | Y | Y | ***N*** | Y | Y | n/a | Y | n/a | Y | ***N*** | n/a | Y(cannabis problems, MCQ, cigarette/day) |
| Wetherill, 2014 | Y | Y | Y | Y | Y | ***N*** | Y | Y | n/a | Y | n/a | Y | ***N*** | n/a | ***N*** |
| Wetherill, 2015 | Y | Y | Y | Y | Y | ***N*** | Y | Y | n/a | Y | n/a | Y | ***N*** | n/a | Y (age, cigarettes & drinks/day, depression) |
| Filbey, 2016 | Y | Y | Y | Y | Y | ***N*** | Y | Y | n/a | Y | n/a | Y | ***N*** | n/a | Y (drinks per drinking day, education years) |
| de Sousa Fernandes Perna, 2017 | Y | Y | Y | Y | Y | ***N*** | Y | Y | n/a | Y | n/a | Y | ***N*** | n/a | ***N*** |
| Vingerhoets, 2016 | Y | Y | Y | Y | Y | ***N*** | Y | Y | n/a | Y | n/a | Y | ***N*** | Y | ***Y*** (baseline CUDIT, AUDIT, FTND, MCQ, cigarettes/day, lifetime use of other psychotropic substances) |
| Wetherill, 2016 | Y | Y | Y | Y | Y | ***N*** | Y | Y | n/a | Y | n/a | Y | ***N*** | n/a | ***N*** |
| Karoly, 2019 | Y | Y | Y | Y | Y | ***N*** | Y | Y | n/a | Y | n/a | Y | ***N*** | n/a | ***N*** |
| Zhou, 2019 | Y | Y | Y | Y | Y | ***N*** | Y | Y | n/a | Y | n/a | Y | ***N*** | n/a | Y (abstinence duration, age of onset) |
| Kuhns, 2020 | Y | Y | Y | Y | Y | ***N*** | Y | Y | n/a | Y | n/a | Y | ***N*** | n/a | Y (alcohol dependence) |
| Yoo, 2020 | Y | Y | Y | Y | Y | ***N*** | Y | Y | n/a | Y | n/a | Y | ***N*** | n/a | Y (age) |
| Kleinhans, 2020 | Y | Y | Y | Y | Y | ***N*** | Y | Y | n/a | Y | n/a | Y | ***N*** | n/a | ***N*** |
| Note: 1.Was the research question or objective in this paper clearly stated?; 2. Was the study population clearly specified and defined?; 3. Was the participation rate of eligible persons at least 50%?; 4a. Were all the subjects selected or recruited from the same or similar populations (including the same time period)? 4b. Were inclusion and exclusion criteria for being in the study prespecified and applied uniformly to all participants?; 5. Was a sample size justification, power description, or variance and effect estimates provided?; 6. For the analyses in this paper, were the exposure(s) of interest measured prior to the outcome(s) being measured?; 7. Was the timeframe sufficient so that one could reasonably expect to see an association between exposure and outcome if it existed?; 8. For exposures that can vary in amount or level, did the study examine different levels of the exposure as related to the outcome (e.g. categories of exposure, or exposure measured as continuous variable)?; 9. Were the exposure measures (independent variables) clearly defined, valid, reliable, and implemented consistently across all study participants?; 10. Was the exposure(s) assessed more than once over time?; 11. Were the outcome measures (dependent variables) clearly defined, valid, reliable, and implemented consistently across all study participants?; 12. Were the outcome assessors blinded to the exposure status of participants?; 13. Was loss to follow-up after baseline 20% or less?; 14. Were key potential confounding variables measured and adjusted statistically for their impact on the relationship between exposure(s) and outcome(s)? Note: Item 8. was deemed ‘not applicable’ as measuring cannabis cues as a dichotomous variable (i.e. cannabis versus neutral cues) was a key inclusion criteria.  Note: Item 10. was deemed ‘not applicable’ as single trials in fMRI analysis are considered a non-reliable method and requires multiple trials for a single reliable measure of cue-reactivity.  Note: Item 13. was deemed ‘not applicable’ as all studies (but one) were cross-sectional. | | | | | | | | | | | | | | | |

| **Section and Topic** | **Item #** | **Checklist item** | **Location where item is reported** |
| --- | --- | --- | --- |
| **TITLE** | | |  |
| Title | 1 | Identify the report as a systematic review. | Page 1 |
| **ABSTRACT** | | |  |
| Abstract | 2 | See the PRISMA 2020 for Abstracts checklist. | n/a |
| **INTRODUCTION** | | |  |
| Rationale | 3 | Describe the rationale for the review in the context of existing knowledge. | Page 5-6 |
| Objectives | 4 | Provide an explicit statement of the objective(s) or question(s) the review addresses. | Page 5-6 |
| **METHODS** | | |  |
| Eligibility criteria | 5 | Specify the inclusion and exclusion criteria for the review and how studies were grouped for the syntheses. | Page 6-7 |
| Information sources | 6 | Specify all databases, registers, websites, organisations, reference lists and other sources searched or consulted to identify studies. Specify the date when each source was last searched or consulted. | Page 6-7 |
| Search strategy | 7 | Present the full search strategies for all databases, registers and websites, including any filters and limits used. | Page 6 |
| Selection process | 8 | Specify the methods used to decide whether a study met the inclusion criteria of the review, including how many reviewers screened each record and each report retrieved, whether they worked independently, and if applicable, details of automation tools used in the process. | Page 6-7 |
| Data collection process | 9 | Specify the methods used to collect data from reports, including how many reviewers collected data from each report, whether they worked independently, any processes for obtaining or confirming data from study investigators, and if applicable, details of automation tools used in the process. | Page 6-7 |
| Data items | 10a | List and define all outcomes for which data were sought. Specify whether all results that were compatible with each outcome domain in each study were sought (e.g. for all measures, time points, analyses), and if not, the methods used to decide which results to collect. | Page 6-7 |
|  | 10b | List and define all other variables for which data were sought (e.g. participant and intervention characteristics, funding sources). Describe any assumptions made about any missing or unclear information. | Page 6-8 |
| Study risk of bias assessment | 11 | Specify the methods used to assess risk of bias in the included studies, including details of the tool(s) used, how many reviewers assessed each study and whether they worked independently, and if applicable, details of automation tools used in the process. | Page 8 |
| Effect measures | 12 | Specify for each outcome the effect measure(s) (e.g. risk ratio, mean difference) used in the synthesis or presentation of results. | n/a |
| Synthesis methods | 13a | Describe the processes used to decide which studies were eligible for each synthesis (e.g. tabulating the study intervention characteristics and comparing against the planned groups for each synthesis (item #5)). | Page 6-7 |
|  | 13b | Describe any methods required to prepare the data for presentation or synthesis, such as handling of missing summary statistics, or data conversions. | Page 8-14 |
|  | 13c | Describe any methods used to tabulate or visually display results of individual studies and syntheses. | Page 22-32 |
|  | 13d | Describe any methods used to synthesize results and provide a rationale for the choice(s). If meta-analysis was performed, describe the model(s), method(s) to identify the presence and extent of statistical heterogeneity, and software package(s) used. | n/a |
|  | 13e | Describe any methods used to explore possible causes of heterogeneity among study results (e.g. subgroup analysis, meta-regression). | n/a |
|  | 13f | Describe any sensitivity analyses conducted to assess robustness of the synthesized results. | n/a |
| Reporting bias assessment | 14 | Describe any methods used to assess risk of bias due to missing results in a synthesis (arising from reporting biases). | Page 8 |
| Certainty assessment | 15 | Describe any methods used to assess certainty (or confidence) in the body of evidence for an outcome. | Page 8 |
| **RESULTS** | | |  |
| Study selection | 16a | Describe the results of the search and selection process, from the number of records identified in the search to the number of studies included in the review, ideally using a flow diagram. | Page 7 & 32 |
|  | 16b | Cite studies that might appear to meet the inclusion criteria, but which were excluded, and explain why they were excluded. | no |
| Study characteristics | 17 | Cite each included study and present its characteristics. | Page 8 & 23 & Supp Material |
| Risk of bias in studies | 18 | Present assessments of risk of bias for each included study. | Supp Material |
| Results of individual studies | 19 | For all outcomes, present, for each study: (a) summary statistics for each group (where appropriate) and (b) an effect estimate and its precision (e.g. confidence/credible interval), ideally using structured tables or plots. | n/a |
| Results of syntheses | 20a | For each synthesis, briefly summarise the characteristics and risk of bias among contributing studies. | Page 8-14 |
|  | 20b | Present results of all statistical syntheses conducted. If meta-analysis was done, present for each the summary estimate and its precision (e.g. confidence/credible interval) and measures of statistical heterogeneity. If comparing groups, describe the direction of the effect. |  |
|  | 20c | Present results of all investigations of possible causes of heterogeneity among study results. |  |
|  | 20d | Present results of all sensitivity analyses conducted to assess the robustness of the synthesized results. |  |
| Reporting biases | 21 | Present assessments of risk of bias due to missing results (arising from reporting biases) for each synthesis assessed. |  |
| Certainty of evidence | 22 | Present assessments of certainty (or confidence) in the body of evidence for each outcome assessed. |  |
| **DISCUSSION** | | |  |
| Discussion | 23a | Provide a general interpretation of the results in the context of other evidence. | Page 14-16 |
|  | 23b | Discuss any limitations of the evidence included in the review. | Page 17-18 |
|  | 23c | Discuss any limitations of the review processes used. | Page 17 |
|  | 23d | Discuss implications of the results for practice, policy, and future research. | Page 18-19 |
| **OTHER INFORMATION** | | |  |
| Registration and protocol | 24a | Provide registration information for the review, including register name and registration number, or state that the review was not registered. | Page 3 & 6 |
|  | 24b | Indicate where the review protocol can be accessed, or state that a protocol was not prepared. | n/a |
|  | 24c | Describe and explain any amendments to information provided at registration or in the protocol. | n/a |
| Support | 25 | Describe sources of financial or non-financial support for the review, and the role of the funders or sponsors in the review. | Page 2 |
| Competing interests | 26 | Declare any competing interests of review authors. | Page 2 |
| Availability of data, code and other materials | 27 | Report which of the following are publicly available and where they can be found: template data collection forms; data extracted from included studies; data used for all analyses; analytic code; any other materials used in the review. | Page 6 |

# References

Beck AT, Steer RA, Brown GK (1996) Beck Depression Inventory-II. The Psychological Corporation, San Antonio, TX

Bitter SM et al. (2014) Neurofunctional changes in adolescent cannabis users with and without bipolar disorder Addiction (Abingdon, England) 109:1901-1909 doi:10.1111/add.12668

Charboneau EJ et al. (2013) Cannabis cue-induced brain activation correlates with drug craving in limbic and visual salience regions: Preliminary results Psychiatry Research: Neuroimaging 214:122-131 doi:10.1016/j.pscychresns.2013.06.005

Cousijn J, Goudriaan AE, Ridderinkhof KR, Van Den Brink W, Veltman DJ, Wiers RW (2013) Neural responses associated with cue-reactivity in frequent cannabis users Addiction Biology 18:570-580 doi:10.1111/j.1369-1600.2011.00417.x

de Sousa Fernandes Perna EB et al. (2017) Brain reactivity to alcohol and cannabis marketing during sobriety and intoxication Addict Biol 22:823-832 doi:10.1111/adb.12351

Feldstein Ewing SW, McEachern AD, Yezhuvath U, Bryan AD, Hutchison KE, Filbey FM (2013) Integrating brain and behavior: Evaluating adolescents’ response to a cannabis intervention Psychology of Addictive Behaviors 27:510-525 doi:10.1037/a0029767

Filbey FM, Dunlop J (2014) Differential reward network functional connectivity in cannabis dependent and non-dependent users Drug Alcohol Depend 140:101-111 doi:10.1016/j.drugalcdep.2014.04.002

Filbey FM et al. (2016) fMRI study of neural sensitization to hedonic stimuli in long‐term, daily cannabis users Human brain mapping 37:3431-3443 doi:10.1002/hbm.23250

Filbey FM, Schacht JP, Myers US, Chavez RS, Hutchison KE (2009) Marijuana craving in the brain Proceedings of the National Academy of Sciences of the United States of America 106:13016-13021 doi:10.1073/pnas.0903863106

Gellermann LW (1933) Chance orders of alternating stimuli in visual discrimination experiments The journal of genetic psychology 42:206-208

Goldman M et al. (2013) Reward-related brain response and craving correlates of marijuana cue exposure: A preliminary study in treatment-seeking marijuana-dependent subjects Journal of Addiction Medicine 7:8-16 doi:10.1097/ADM.0b013e318273863a

Karoly HC, Schacht JP, Meredith LR, Jacobus J, Tapert SF, Gray KM, Squeglia LM (2019) Investigating a novel fMRI cannabis cue reactivity task in youth Addictive behaviors 89:20-28 doi:10.1016/j.addbeh.2018.09.015

Lang PJ, Bradley M, Cuthbert B (1988) The international affective picture system Center for Research in Psychophysiology, University of florida, Gainesville, USA

Marchewka A, Żurawski Ł, Jednoróg K, Grabowska A (2014) The Nencki Affective Picture System (NAPS): Introduction to a novel, standardized, wide-range, high-quality, realistic picture database Behavior Research Methods 46:596-610 doi:10.3758/s13428-013-0379-1

McClernon FJ, Hiott FB, Huettel SA, Rose JE (2005) Abstinence-induced changes in self-report craving correlate with event-related FMRI responses to smoking cues Neuropsychopharmacology : official publication of the American College of Neuropsychopharmacology 30:1940-1947 doi:10.1038/sj.npp.1300780

Sobell LC, Sobell MB (1992) Timeline Follow-Back. In: Litten RZ, Allen JP (eds) Measuring Alcohol Consumption: Psychosocial and Biochemical Methods. Humana Press, Totowa, NJ, pp 41-72. doi:10.1007/978-1-4612-0357-5_3

Vingerhoets W et al. (2016) Cue-induced striatal activity in frequent cannabis users independently predicts cannabis problem severity three years later Journal of Psychopharmacology 30:152-158 doi:10.1177/0269881115620436

Wetherill RR et al. (2014) Neural responses to subliminally presented cannabis and other emotionally evocative cues in cannabis-dependent individuals Psychopharmacology 231:1397-1407 doi:10.1007/s00213-013-3342-z

Wetherill RR, Hager N, Jagannathan K, Mashhoon Y, Pater H, Childress AR, Franklin TR (2016) Early Versus Late Onset of Cannabis Use: Differences in Striatal Response to Cannabis Cues Cannabis and Cannabinoid Research 1:229-233 doi:10.1089/can.2016.0026

Wetherill RR, Jagannathan K, Hager N, Childress AR, Franklin TR (2015) Sex differences in associations between cannabis craving and neural responses to cannabis cues: Implications for treatment Experimental and Clinical Psychopharmacology 23:238-246 doi:10.1037/pha0000036

Zhou X et al. (2019) Cue reactivity in the ventral striatum characterizes heavy cannabis use, whereas reactivity in the dorsal striatum mediates dependent use. Biological psychiatry Cognitive neuroscience and neuroimaging doi:10.1016/j.bpsc.2019.04.006
